# Potential use of the Asteraceae family as a cure for diabetes: A review of ethnopharmacology to modern day drug and nutraceuticals developments

**DOI:** 10.3389/fphar.2023.1153600

**Published:** 2023-08-03

**Authors:** Yugal Kishore Mohanta, Awdhesh Kumar Mishra, Amilia Nongbet, Ishani Chakrabartty, Saurov Mahanta, Bhaskar Sarma, Jibanjyoti Panda, Sujogya Kumar Panda

**Affiliations:** ^1^ Nano-biotechnology and Translational Knowledge Laboratory, Department of Applied Biology, School of Biological Sciences, University of Science and Technology Meghalaya (USTM), Techno City, Meghalaya, India; ^2^ Centre for Herbal Pharmacology and Environmental Sustainability, Chettinad Hospital and Research Institute, Chettinad Academy of Research and Education, kelambakkam, Tamil Nadu, India; ^3^ Department of Biotechnology, Yeungnam University, Gyeongsan, Republic of Korea; ^4^ Department of Botany, School of Biological Sciences, University of Science and Technology Meghalaya (USTM), Techno City, Meghalaya, India; ^5^ Learning and Development Solutions, Indegene Pvt. Ltd., Manyata Tech Park, Bangalore, India; ^6^ Guwahati Centre, National Institute of Electronics and Information Technology (NIELIT), Guwahati, Assam, India; ^7^ Department of Botany, Dhemaji College, Dhemaji, Assam, India; ^8^ Center of Environment Climate Change and Public Health, RUSA 2.0, Utkal University, Bhubaneswar, Odisha, India

**Keywords:** Asteraceae, diabetes, drug development, DiaNat-DB, ethnomedicine, nutraceuticals

## Abstract

The diabetes**-**associated mortality rate is increasing annually, along with the severity of its accompanying disorders that impair human health. Worldwide, several medicinal plants are frequently urged for the management of diabetes. Reports are available on the use of medicinal plants by traditional healers for their blood-sugar-lowering effects, along with scientific evidence to support such claims. The Asteraceae family is one of the most diverse flowering plants, with about 1,690 genera and 32,000 species. Since ancient times, people have consumed various herbs of the Asteraceae family as food and employed them as medicine. Despite the wide variety of members within the family, most of them are rich in naturally occurring polysaccharides that possess potent prebiotic effects, which trigger their use as potential nutraceuticals. This review provides detailed information on the reported Asteraceae plants traditionally used as antidiabetic agents, with a major focus on the plants of this family that are known to exert antioxidant, hepatoprotective, vasodilation, and wound healing effects, which further action for the prevention of major diseases like cardiovascular disease (CVD), liver cirrhosis, and diabetes mellitus (DM). Moreover, this review highlights the potential of Asteraceae plants to counteract diabetic conditions when used as food and nutraceuticals. The information documented in this review article can serve as a pioneer for developing research initiatives directed at the exploration of Asteraceae and, at the forefront, the development of a botanical drug for the treatment of DM.

## 1 Introduction

Diabetes mellitus (DM), or insulin-dependent diabetes, is a metabolic disorder in which the blood glucose level increases above the normal threshold (Ansari et al., 2020; Ansari et al., 2021a). In this condition, the β-cells of the islets of Langerhans of the pancreas are either unable to produce insulin or their glucose-utilizing ability is inhibited. In some cases, the cells may resist insulin uptake and utilization. This disorder is a chronic illness with a global social and economic impact (Ansari et al., 2022b). Low insulin levels affect several tissues, like skeletal muscles, adipose tissues, the kidneys, and the liver, to such an extent that they downregulate the signal transduction system and the expression of associated genes. Hyperglycemia is often accompanied by several symptoms, including extreme weight loss, polyuria, and polyphagia, and under severe conditions, it can lead to ketoacidosis, which may result in death (Kharroubi and Darwish, 2015). According to the World Health Organization (WHO), around 422 million adults suffered from DM in 2014, and it was the ninth leading cause of death globally in 2019, accounting for approximately 1.5 million deaths (WHO, 2021). Interestingly, DM is a “silent” illness that can worsen due to negligence and insufficient healthcare. Approximately 47% of diabetic individuals are unaware of their condition (Adailton da Silva et al., 2018). As a result, a DM diagnosis is often met with feelings of shock and denial, which further leads to a reluctance to undergo treatment involving important lifestyle-related changes. Many synthetic drugs are available for managing and treating type-2 DM (DM2); however, they often fail to address the complications of the disease, and most available therapies seem insufficient. Most patients require regular insulin administration, and around 75% develop CVD (Goje et al., 2014; Jayaweera et al., 2022).

**FIGURE 1:**
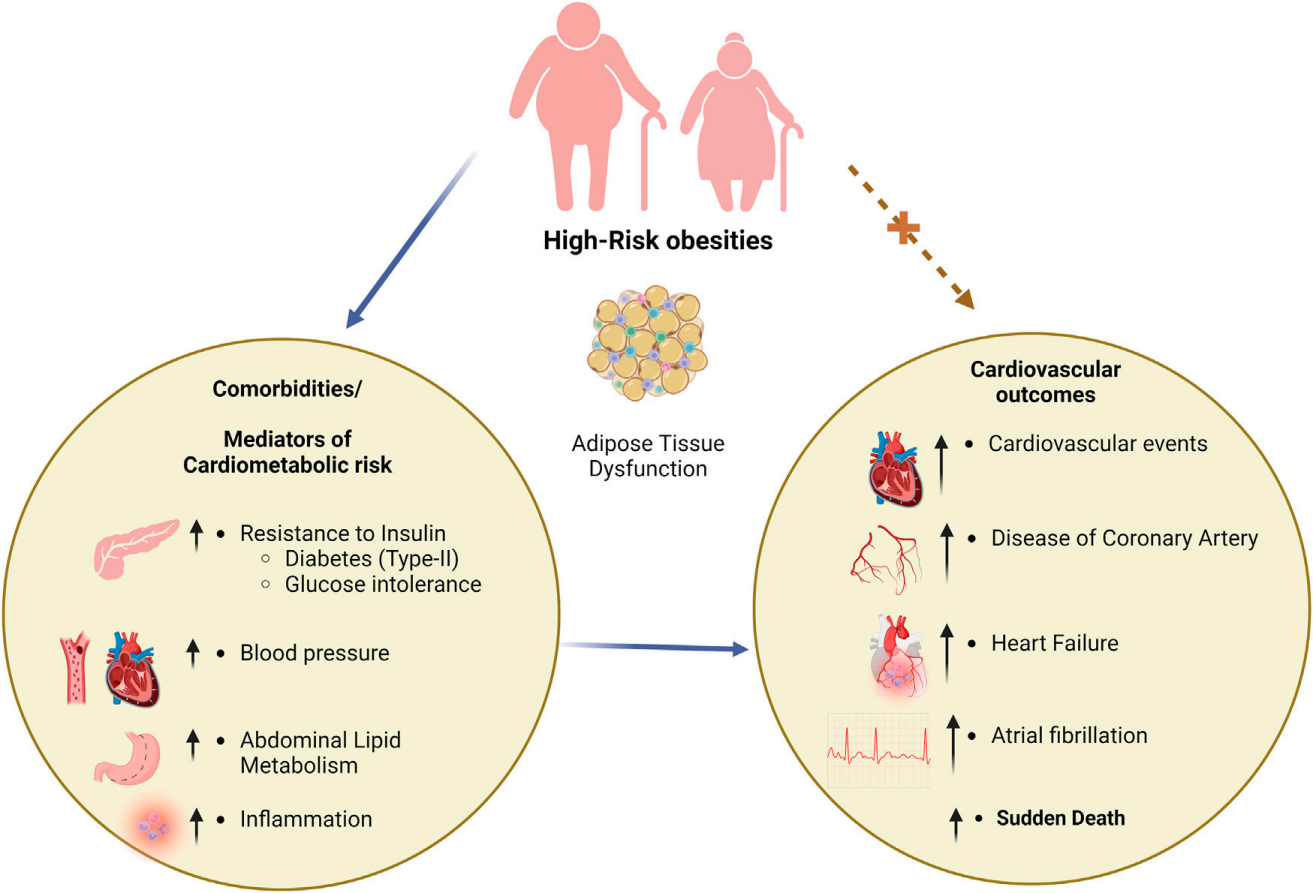
Correlation between obesity, DM, and CVD. DM; diabetes mellitus, CVD; cardiovascular disease.

Obesity and DM2 have long been linked, which explains the high prevalence of DM2 in many developed countries, with DM2 also being a substantial risk factor for CVD (Piché et al., 2020; Ansari et al., 2021c). Because obesity is frequently associated with hypertension and dyslipidemia, many high-risk obese patients have a confluence of metabolic and cardiovascular risk factors. Thus, obesity (especially in its high-risk forms) is a standard driver of altered metabolic activities that lead to DM and cardiovascular risk factors that can be targeted with pharmacotherapies (Flegal et al., 2007). The relationships between obesity and high-risk cardiometabolic risk variables, such as DM, and the consequences of cardiovascular disease have been depicted in Figure 1.

The prevalence of obesity is a risk factor for developing DM2 (Flegal et al., 2007). Numerous studies using upper body, abdominal adiposity anthropometric indices, or direct imaging measurements (CT or MRI) have shown that an enlarged waistline, a higher waist-to-hip ratio, or higher levels of visceral adipose tissue (VAT) at any body mass index (BMI) level significantly raise the risk of developing DM2, in addition to overall obesity (Després, 2012). Patients with DM2 tend to be overweight and have a higher VAT than those with a similar body mass index who do not have DM. While having each of these conditions individually increases the risk of cardiovascular consequences, having both visceral or severe obesity and DM2 should exponentially increase that risk. The enhanced risk of developing cardiovascular problems and heart failure due to the increasingly common coexistence of DM2 and high-risk forms of obesity poses complex management issues. Insulin resistance, which may be partially mediated by obesity, DM2, and cardiovascular risk, is associated with ectopic and visceral adiposity. CVD mortality risk has been linked to metabolic syndrome and insulin resistance (Li et al., 2007).

The lack of available treatments and economical insufficiency to procure expensive treatments have led scientists and researchers to resort to alternative medicine. As such, botanical drugs, herbal medicines, and other dietary supplements may be suitable for treating DM.

In the present stressful world, a balanced diet and healthy lifestyle are of utmost importance. Foods fortified with high amounts of antioxidants, vitamins, and minerals are gaining tremendous importance. According to Health Line, fortified foods refer to items low in trans-fat but rich in vitamins, minerals, probiotics, fiber, and metabolites with high nutritional value and substantial health benefits (Link, 2020). A prominent issue here is that “nutraceuticals” refer to the active components present in functional foods that can be extracted, purified, and concentrated for use and have different usages apart from being “functional foods.” An online search on PubMed and Google Scholar demonstrates that diet management is an essential parameter for preventing and curing diseases such as CVD, DM, hypertension, obesity, and thyroid disorders (Chakrabartty et al., 2022). A large body of clinical evidence suggests that DM2 and its associated complications can be prevented. The risk of disease can be avoided by the consumption of foods that are rich in antioxidants, have reasonable glycemic control, regulate blood pressure, promote the growth of favorable gut microbes, lower inflammatory cytokine secretion, and activate the production of “good” enzymes and hormones (Alkhatib et al., 2017). It is interesting to note that the traditionally used Mediterranean diet (MD) is considered one of the healthiest diets for human longevity and the prevention of chronic illnesses like DM, as it is rich in secondary metabolites and other components that are recommended for the prevention of DM (Rivera et al., 2010; Mirmiran et al., 2014). Many plant families like Zingiberaceae (e.g., *Alpinia galanga* (L.) Wild.), Lamiaceae (e.g., *Salvia officinalis* L.), Caryophyllaceae (e.g., *Corrigiola litoralis* sub sp. *foliosa* (Pérez Lara) Devesa), and many others have been consumed traditionally for many generations due to their many health benefits (Ansari et al., 2021b; Ansari et., 2022a; Ansari et al., 2022d).

For a long time, plants have had a strong foothold in traditional medicine and form the basis of modern-day medications. Age-old medicine practices like Ayurveda and Unani have practitioners who rely on different plants and their parts to treat various diseases, and these treatment systems are still practiced in Southeast Asia (Ansari et al., 2022a; Ansari et al., 2022c). However, the problem with traditional medicines is the lack of documentation, authenticity, and scientific evidence. Most of the ‘bej, ‘kobiraj,’ or local healers have learned the art and knowledge of traditional healing from their ancestors, having been handed down as a legacy. According to the WHO, most of the global population relies on herbal medicines and drugs to treat diseases, from wound healing to CVD treatments. Interestingly, some of these medicinal plants find their use as one or more culinary spices and are consumed as food. One of the most common examples in this respect is the Zingiberaceae family, the natural ‘pharmacy’ of the plant kingdom and the rich flavoring ingredient of the kitchen. *Alpinia nigra* (Gaertn.) Burtt, a lesser explored member of this family, is traditionally used to treat gut infections caused by *Fasciolis buski* and is also used to prepare delicacies like ‘eromba’ and ‘deragong’ in Manipur and Tripura, respectively (Swargiary et al., 2013; Swargiary and Verma, 2015; Chakrabartty et al., 2019). Asteraceae is one such family of medicinal plants that has not been explored much but houses a wide range of plants to treat diseases (Ansari et al., 2021d).

Asteraceae, or Compositae, also known as the ‘daisy’ family, is a large family of angiosperms that harbors around 32,000 plant species with a cosmopolitan distribution, growing in all regions of the world except Antarctica. These plants have been used as medicines and as part of the human diet for ages. This family includes some well-known medicinal plants like lettuce, chamomile, and dandelion, as well as ornamental plants like daisies, sunflowers, chicory, and dahlias (Aboul Ela et al., 2012; Michel et al., 2020). Some of these plants and their parts are caffeine-free, extremely rich in vitamin C, E, and organic acids, and commonly used in salads and sandwiches. As such, these plants can be widely cultivated as sources of functional foods. Different species of Asteraceae have been reported to exhibit other pharmacological properties that can be attributed to the presence of large quantities of phytochemicals and secondary metabolites, including essential oils, lignans, tannins, flavonoids, alkaloids, phenolic acids, and saponins. Most of these plants have many sesquiterpene lactones, which impart a bitter taste (Djellouli et al., 2013; Rolnik and Olas, 2021). Secondary metabolites are responsible for antioxidant, anticancer, and antimicrobial properties; hence, the plants of the Asteraceae family can be exploited for the treatment of certain serious diseases like CVD, hypertension, and DM.

The present review aims to bridge the gap in existing literature regarding the role of several members of the family Asteraceae, particularly in preventing DM2 and diabetic wounds, and their application as fortified foods. Extensive literature is available on the role of medicinal plants in the management of lifestyle-related disorders. But this review is novel as it focuses explicitly on managing DM2 using different members of the Asteraceae family alone. Not only that, the utilization of secondary metabolites and bioactive compounds from this family as dietary supplements and fortified foods is highlighted, specifically focused on the management of DM. This review is subdivided into different sections: Section 2 describes the importance of this family in ethnopharmacology and traditional medicine. Section 3 highlights the role of the Asteraceae plant metabolites in treating and preventing DM. In Section 4, the role of different members of the Asteraceae in wound healing has been described in detail. Section 5 describes the untapped and identified compounds from Asteraceae against DM. Sections 6 and 7 discuss the prospects of research in Asteraceae (in search of novel bioactive compounds to be used as medicines and fortified foods). The challenges and opportunities to utilize and establish the Asteraceae family plants as medicines and nutraceuticals have also been discussed in Section 8, and the concluding remarks on the present study have been discussed in Section 9.

## 2 Asteraceae: the largest family with diverse medicinal properties

The capacity of plants to produce secondary metabolites with considerable biological activity has long made them an integral part of the evolution of medicine. Traditional medicine relies heavily on plant remedies to address various health issues. Conventional medications derived from plants are readily available to the local population, are economical, and have no side effects, as supported by many years of successful human use. In recent years, more advanced analytical techniques have been developed, identifying several new phytochemicals.

Asteraceae is one of the largest plant families, with 32,000 accepted species names under 1,690 accepted genera (POWO, 2022). A few prominent genera include *Senenio* (1,470 spp.), *Vernonia* (1,050 spp.), *Cousinia* (600 spp.), *Eupatorium* (590 spp.), *Centaurea* (590 spp.), *Hieracium* (470 spp.), *Helichrysum* (460 spp.), *Saussurea* (300 spp.), *Cirsium* (270 spp.), *Aster* (240 spp.), and *Bidens* (210 spp.). Many species are cultivated, including several widely used culinary botanical drugs, viz., *Artemisia annua* L.*, Blumea balsamifera* (L.) DC.*,* and *Calendula officinalis* L.*,* (Rodríguez-Chavez et al., 2017). For centuries, the species of the family Asteraceae have been used worldwide as traditional medicine against various human ailments, including DM, kidney, heart, lung, liver, and skin toothache inflammation, pain, constipation, toothache, throat pain, snake bite, headache, gastrointestinal disorders, diarrhea, dysentery, tuberculosis, hepatitis, asthma, menopausal and menstrual disorders, stomach ulcers, sores, scabies, filariasis, elephantiasis, night-blindness, impotence, hair fall, jaundice, nose bleeding, allergies, viral infections, cough, bronchitis, different types of cancers, wounds and cuts, and malaria (Bessada et al., 2015; Panda and Luyten, 2018; Panda et al., 2019; Michel et al., 2020; Garcia-Oliveira et al., 2021; Woldeamanuel et al., 2022).

*Artemisia* is the other important member of this family, considered to comprise >500 medicinal species in the world (Martín et al., 2003; Bisht et al., 2021). Traditionally, the members of *Artemisia* have been used to treat several diseases like hepatitis, cancer, inflammation, DM, and infections caused by microorganisms (Asad et al., 2011). The discovery of artemisinin, which resulted in the 2015 Nobel Prize in Physiology or Medicine, has revolutionized the treatment of malaria worldwide (Panda and Luyten, 2018).

The *Achillea* genus is well known for its use in traditional medicine (Mohammadhosseini et al., 2017; Jarić et al., 2018). The greatest research has been done on *Achillea millefolium* L. Yarrow, a perennial plant native to the temperate regions of Europe and Asia. It has been utilized by humans for well over 3,000 years (Ali et al., 2017).

The genus *Achyrocline* (distributed across Latin America) is well-known for its medicinal properties (Retta et al., 2012; Bolson et al., 2015). Reports from ethnobotanical studies conducted in the Brazilian state of Rio Grande do Sul showed that the plant *Achyrocline satureioides* (Lam.) DC. is often used as a healing agent (Carvalho et al., 2018).

As early as the 13th century, Europeans used the *C. officinalis* (or calendula) plant to cure wounds. Since then, several cosmetic products have been created using components derived from calendula (Parente et al., 2012). In the middle ages, Calendula flowers were used for liver obstructions, snake bites, and heart strengthening (Cetkovic et al., 2004).

Since ancient times, cardoon (*Cynara cardunculus* L.) has played an essential role in the culinary and medicinal traditions of the Mediterranean. A wide variety of therapeutic properties, such as antioxidant, lipid-lowering, anti-inflammatory, antidiabetic (related to DM), antibacterial, and anticancer abilities, have been attributed to the various bioactive substances of Cardoon (Silva et al., 2022).

*Eclipta prostrata* (L.) L. is a plant that is known by several names in India and is reported to be used as a treatment for skin, respiratory (asthma), and hepatic (jaundice) conditions, gastrointestinal disorders, and spleen enlargement (Dalal et al., 2010; Jahan et al., 2014). In Nepal, people utilize its leaves and young stems to cure wounds and prevent infections (Manandhar, 2002; Adhikari et al., 2019), and many Bangladeshi ethnic groups use it to cure jaundice. Additionally, it is used to treat acidity, alopecia (Roy et al., 2008), gingivitis, fever, bodily aches, asthma, bronchitis, burns, constipation, wounds, wrinkles, edema, pimples, and other skin conditions (Kumari et al., 2006; Tewtrakul et al., 2007; Khan and Khan, 2008; Neeraja and Margaret, 2011).

Numerous communities have recognized and used the medicinal properties of the plants in the genus *Pluchea* (Gridling et al., 2009; Schmidt et al., 2009; Ab Rahman et al., 2014).

*Solidago virgaurea* L., European goldenrod (or woundwort), is a well-studied common medicinal plant across Europe and other areas. European goldenrod has been used for centuries to cure diseases of the urogenital tract (Toiu et al., 2019). In different parts of the world, its infusion or decoction is used in traditional medicine to treat bacterial infections and inflammation (Gross et al., 2002).

For a long time of people using *Taraxacum* sp. (dandelion) as a therapeutic herb. It is used as a food item in many countries for preventing and treating DM2. Because of its hepatic and hyperglycemic properties, dandelion has been used traditionally as a folk medicine in Russia, China, and India (Kemper, 1999). It is often used as a meal (in salads) because of its high vitamin and mineral content (Escudero et al., 2003). Dandelion is a popular traditional medicine in Turkey and Mexico used to manage DM2 (Onal et al., 2005).

*Xanthium strumarium* L. has been used as a medicine in China for centuries, and it is one of the most widely used herbal medications for treating rhinitis and headaches. In addition, *X. strumarium* has been documented as a traditional herbal remedy for various health issues in Bangladesh, including urinary tract infections, ear infections, DM, and indigestion (Islam et al., 2009).

*Gundelia tournefortti* L. is a plant with seeds that is often used to make pickles and acts as a diuretic (Çoruh et al., 2007). Traditional Brazilian medicine prescribes it for renal and cardiac ailments. Clinical research found that the anticoagulant properties of *Emilia praetermissa* Milne-Redh. helped improve hyperlipidemic disorders (Memariani et al., 2018). Another plant with significant ethnopharmacological value for wound healing is *Silybum marianum* (L.) Gaertn (Hudaib et al., 2008; Aziz et al., 2016; Tabari et al., 2019).

### 2.1 Asteraceae with particular references to traditional uses in the treatment of DM

The Asteraceae family is the group of plants that have been traditionally used to treat DM (Table 1) and has received the greatest attention. However, not all of them have been thoroughly validated by scientists. This includes *Artemisia afra* Jacq. ex Willd., *Brachylaena elliptica* (Thunb.) Less, *Brachylaena discolor* DC, *Helichrysum gymnocomum* DC, *Helichrysum nudifolium* (L.) Less, *Helichrysum odoratissimum* (L.) Sweet., and *Helichrysum petiolare* Hilliard & B. L. Burtt., *Conyza scabrida* DC., *Schkuhria pinnata* (Lam.) Kuntze ex Thell., *Tarchonanthus camphoratus* L., *Pteronia divaricata* (P. Bergius) Less., *Vernonia oligocephala* Katt., and *Vernonia amygdalina* Delile (Odeyemi and Bradley, 2018). A fair number of plants in this family have been shown to have a variety of effects on the body, including lowering blood sugar levels, increasing insulin production, repairing damaged pancreatic β-cells, inhibiting carbohydrate digesting enzymes, and protecting against oxidative stress (Gyang et al., 2005; Osinubi, 2006; Atangwho et al., 2008, 2013; Ebong et al., 2008; Okolie et al., 2008; Deutschländer et al., 2009; Afolayan and Sunmonu, 2010; van Huyssteen et al., 2011; Wintola and Afolayan, 2011; Mellem, 2013; Sunmonu and Afolayan, 2013; Mellem et al., 2015). Most of the anti-diabetic molecules retrieved from these plants represented saponins, flavanones, tannins, and flavonoids (aglycones) (Mukherjee et al., 2006; van Huyssteen et al., 2011; Sunmonu and Afolayan, 2013).

**TABLE 1:** Asteraceae plants used traditional as anti-diabetic botanical preparation.

Botanical Name	Parts used	Traditional uses	References
*Achillea asiatica S*erg	Wp	DM	Dorjsembe et al. (2017)
*Achyrocline alata* (Kunth) DC.	Wp	DM	Pereira et al. (2017)
*Acmella oleracea* (L.) R.K.Jansen	Wp	DM, throat pain, stomach trouble, toothache, snake bite, pain reliever, fungal infection, cancer	Wu et al. (2008), Buragohain (2011), Abeysiri et al. (2013), Dubey et al. (2013), Maria-Ferreira et al. (2014), Gerbino et al. (2016)
*Ageratum conyzoides* (L.) L	Lf	DM, fever, rheumatism, cardiovascular	Agbafor et al. (2015)
		Malaria, wounds, spasms	
*Artemisia princeps* Pamp.	Wp	DM	Lee et al. (2014)
*Artemisia capillaris* Thunb.	Wp	Hepatitis, Inflammation, DM.	Jang et al. (2015)
*Bidens Pilosa* L	Rt	DM, wounds, hepatitis, diarrhea, urinary tract infections, cold, glandular sclerosis	Yang (2014), Xuan and Khanh (2016)
*Blumea balsamifera* (L.) DC.^a^	Lf	DM, wound healing and cancer activity	Adebayo et al. (2010), Pang et al. (2017)
*Calendula officinalis* L	Wp	DM, amoebic and bloody dysentery	Dinda et al. (2015)
*Cynara cardunculus* L	Wp	DM	Kuczmannová et al. (2016)
*Eclipta prostrata* (L.) L	Wp	DM, cuts and wound, bleeding, leucorrhea, hair fall. Jaundice, diarrhea, malaria activities	Alam and Sharma (2020)
*Galinsoga parviflora* Cav.	Lf, St	Fever, diarrhea, cuts and wound, DM, microbial infections	Mostafa et al. (2013)
*Inula cappa* (Buch.-Ham. ex D. Don) DC^a^	Lf	DM and jaundice	Rai and Lalramnghinglova (2011)
*Lactuca sativa* L	Lf	DM	Chadchan et al. (2016), Ismail et al. (2019), Nabi and Abdullah (2020)
*Silybum marianum* (L.) Gaertn	Fd, Sd	Alcoholic induced liver diseases, DM, viral infection, inflammation, wound healing, cytotoxicity	Anderson et al. (1994), Skottová and Krecman (1998), Hudaib et al. (2008), Jadhav and Upasani (2011), Aziz et al. (2016)
*Solidago virgaurea* L	Ap	Anti-inflammatory, spasmolytic, kidney and bladder inflammation, urolithiasis, cystitis, DM, allergies, and gastro-intestinal disorders	Móricz et al. (2016) (2020), Toiu et al. (2019)
*Taraxacum officinale* (L.) Weber ex F.H.Wigg.	Wp	DM	Schütz et al. (2006), González-Castejón et al. (2012)
*Taraxacum platycarpum* Dahlst.	Wp	DM and hepatic diseases	Petlevski et al. (2003)
*Tithonia diversifolia* (Hemsl.) A.Gray^a^	Fl	DM, stomach pains, indigestion, sore throat, liver pains, wounds and bruises, inflammation, diarrhea	Baruah et al. (1979), Rüngeler et al. (1998), Tona et al. (1998), Tona et al. (1999), Tona et al. (2000)
*Vernonia amygdalina* Delile^a^	Lf	DM, gastrointestinal disorders, amoebic dysentery, malaria, helminth infections	Asante et al. (2016), Oyeyemi et al. (2018)
*Xanthium strumarium* L	Wp	DM, filariasis, chronic malaria, treatment of leukoderma, deadly insect bites, epilepsy, and biliousness	Chopra et al. (1986), Hsu et al. (2000)

^a^ Shurbs, @ plant and all other species are herbs; Wp-Whole plant, Lf-Leaves, Fd- Flower head, St-Stem, Rt-Root, Ap- Aerial part, Fl-flower, Sd-Seed. DM; diabetes mellitus.

Several Asteraceae plants have also been reported, in Indian Ayurvedic medicines, e.g., *Eclipta alba* for skin diseases (Islam et al., 2012), *Ageratum conyzoides, Artemisia capillaries,* and *Inula cappa* for DM (Rai and Lalramnghinglova, 2011; Jang et al., 2015). The decoction of whole plants and roots of *Blumea laciniata* and *B. lacera* has been used to cure bronchitis, blood diseases, fever, burning sensations, and snake bites by the tribes of India (Shukla et al., 2010; Rai and Lalramnghinglova, 2011; Rosangkima and Jagetia, 2015). The extracts of *Ageratum conyzoides* L., *Bidens pilosa* L., and *B. balsamifera* (L.) DC. have been traditionally used as wound-healing agents and cancer treatments in India (Adebayo et al., 2010; Bartolome et al., 2013). Some species of *Vernonia* are most extensively reported as traditional medicine for treating diseases of the skin, central nervous system, kidney, gastrointestinal tract, metabolism, and general health (Toyang and Verpoorte, 2013; Carvalho et al., 2018).

## 3 Asteraceae: undisputable metabolites of interest in DM

The rate of diabetes is increasing globally, including in India, now known as the diabetic capital of the world. Traditional knowledge and the use of medicinal plants to treat severe diseases like DM are emerging again in countries like India because of the fewer side effects and treatment-associated risks. Many members of different plant families like Asteraceae, Zingiberaceae, Lamiaceae, Myrtaceae, and others have been exploited for their antidiabetic properties (Bindu and Narendhirakannan, 2019; Chakrabartty et al., 2020; Michel et al., 2020). Therefore, this review focused on studying the Asteraceae family. In the subsequent sections, five major anti-diabetic plants belonging to this family and their mechanisms of action are discussed in detail.

### 3.1 *Taraxacum officinale* (L.) Weber ex F.H.Wigg. (dandelion)

**FIGURE 2:**
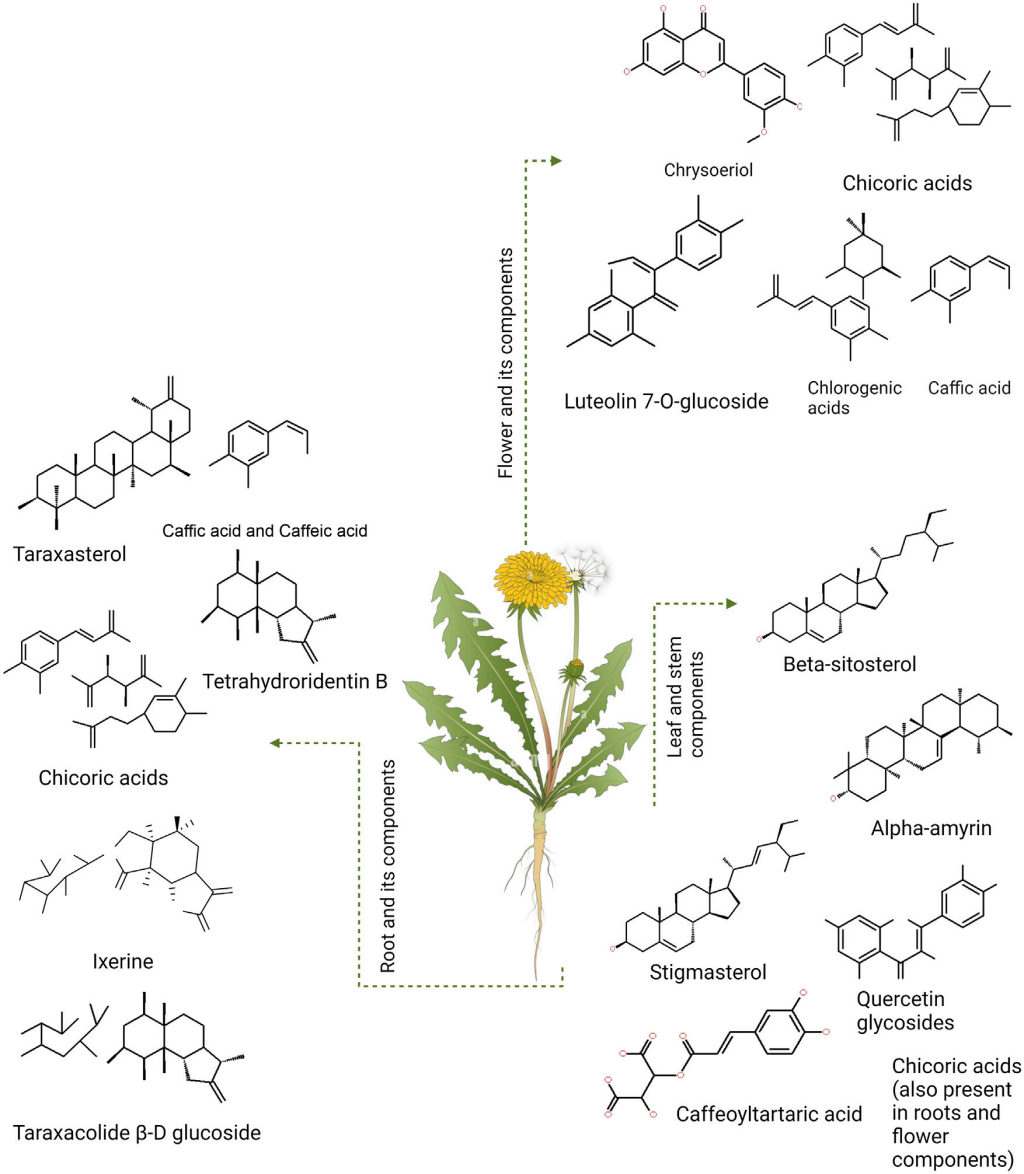
*Taraxacum officinale* (dandelion) and the components present different parts of the plant (flowers, stems, and leaves).

Dandelion is a folk medicine in Russia, India, and China for its hepatic and hyperglycemic properties (Wirngo et al., 2016). In many parts of the world, it is utilized as food and, sometimes, as a treatment approach for DM. This plant has bioactive compounds, such as chicoric acid, taraxasterol (TS), chlorogenic acid, and sesquiterpene lactones, that carry an anti-diabetic potential (Figure 2) (Schütz et al., 2006; González-Castejón et al., 2012). The root of the dandelion contains inulin, which includes fructooligosaccharides (FOS), and can help normalize blood sugar levels and reduce hyperglycemia. It also impacts insulin secretion and sensitivity, making it an attractive option for use as an anti-diabetic drug (Salehi et al., 2019).

Insulin resistance is the primary cause of hyperglycemia and a hallmark of DM2 pathogenesis (Hamden et al., 2008). Another well-known mechanism that affects glucose homeostasis is oxidative stress, which is driven by auto-oxidation and protein glycation (Giugliano et al., 1996). Studies have shown that dandelion extract may stimulate the release of insulin in pancreatic β-cells (Figure 2), which subsequently counteracts the effects of hyperglycosemia (Hussain et al., 2004).

**FIGURE 3:**
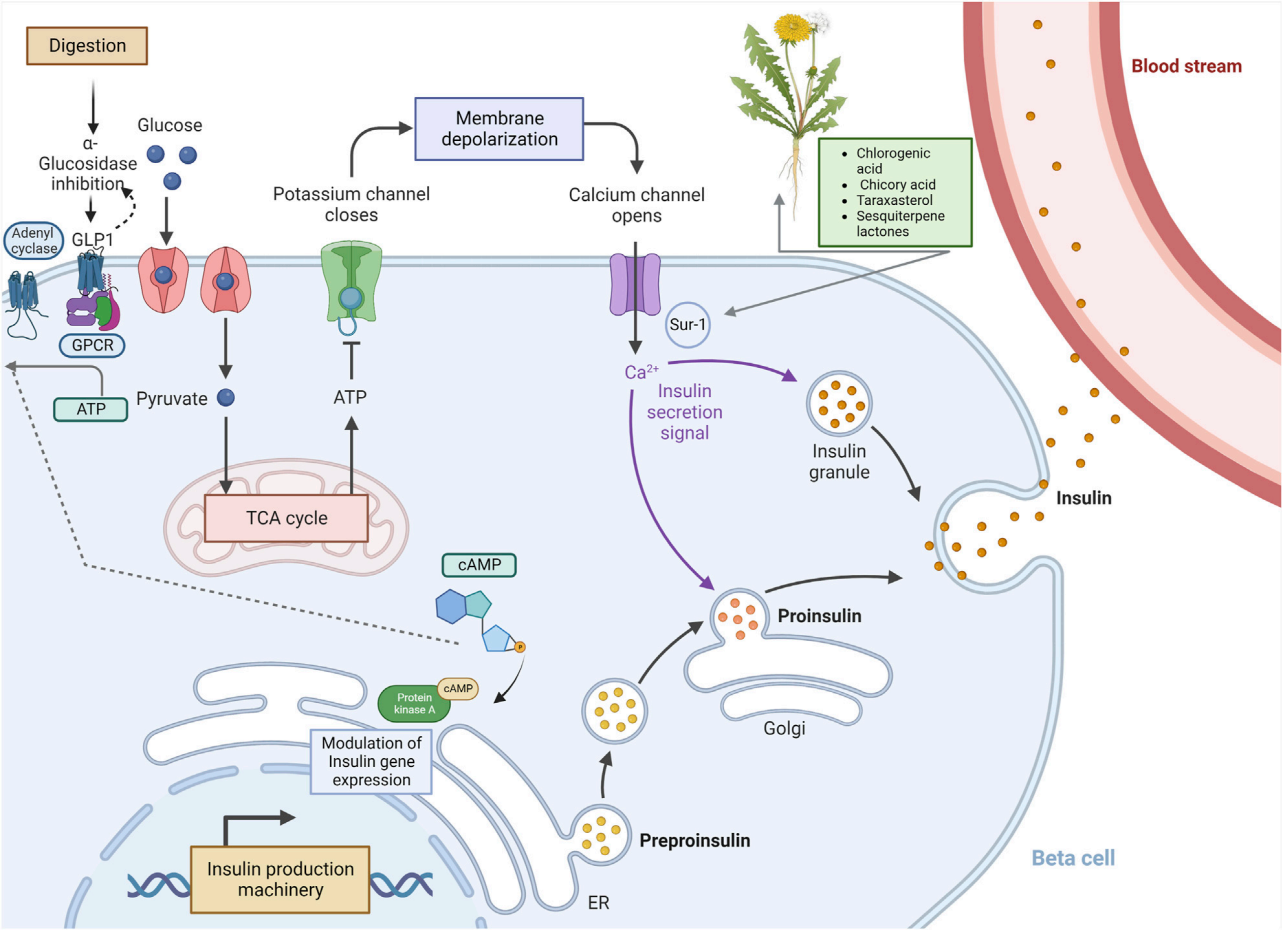
Potential biological mechanisms of *Taraxacum officinale* (dandelion)-derived compounds that modulate cAMP and insulin secretion. The diagram specifies the potential means of bioactive components from *T. officinale* (CGA, CRA, TS & SEL) on insulin secretion and the c-AMP pathway. The DM2 can give rise to several defects, including low response in β-cells (at the receptor or transportation platforms such as GULT2, SUR1, G-protein coupled receptor, and gene expression), glycolysis, Krebs^’^ cycle, and enzymatic digestion. Bioactive components may act directly or indirectly in a series of processes, thereby modulating and regulating DM2 defects and responses. DM2: diabetes mellitus type 2.

Dandelion extract exerts anti-hyperglycemic properties in non-obese diabetic mice (Petlevski et al., 2003). Chicory acid (CRA) stimulates pancreatic insulin production, which enhances glucose absorption in muscle cells (Tousch et al., 2008). CRA and TS block α-glucosidase and α-amylase, preventing the digestion of complex carbohydrates like starch. In diabetic mice, dandelion reduces plasma glucose levels, improving β-cell insulin secretion (Schütz et al., 2006). Chlorogenic acid (CGA), CRA, taraxasterol (TS), and sesquiterpene lactones (SEL) (Figure 3) have considerable promise as DM regulators. Research suggests that TS may be the strongest regulator of DM (Akashi et al., 1994; Williams et al., 1996).

DM treatments produced from medicinal plants could potentially be more cost-effective and accessible after extensive study. At the same time, research into the effects and mechanisms of action of dandelion’s bioactive components has previously been conducted *in vitro* and *in vivo*. Identifying the specific bioactive components of the plant and the associated mechanisms of action in DM requires *in vitro* studies on clonal β-cell (INS-1E), α-cell, and human skeletal cell lines. As a result, this study can yield new information for improving diabetic treatment, which will undoubtedly lessen the global societal and economic burden caused by DM (Wirngo et al., 2016).

The efficacy and safety of the dandelion extract as a treatment for DM2 might be easily studied with clinical trials; however, those are still missing while there is clinical trial data available on the effect of this extract on other conditions such as eczema and migraines ( *https://clinicaltrials.gov/ct2/results?cond=Taraxacum+officinale+&term=&cntry=&state=&city=&dist=* ). Moreover, the bioavailability and metabolism of the individual components of the dandelion extract necessitate more studies.

### 3.2 *Cynara cardunculus* L. (Cardoon)

Abnormal blood glucose levels due to a deficiency in insulin secretion and/or insulin activity characterize DM. *C. cardunculus* has been studied by numerous scientists for its anti-diabetic properties and putative mechanisms of action. Ben Salem et al. (2017) tested the *in vitro* α-amylase inhibitory potential of *C. cardunculus,* and the ethanolic extract was found to be the most potent, with an IC_50_ value of 72.22 ug/mL (higher than that of an acarbose-specific inhibitor, which was 14.83 ug/mL). Furthermore, the scientists tested the ethanol leaf extract of *C. cardunculus* under alloxan-induced stress in Wistar rats to see if it had any anti-diabetic effects. For 28 days, Wistar rats were given 200 or 400 mg/kg of artichoke leaf extract (ALE) as a dietary supplement. When given to diabetic rats, ALE reduced their serum α-amylase levels, reducing their blood glucose rate. Additionally, diabetic rat lipid profiles and antioxidant activity were impacted by ALE treatment. Moreover, Kuczmannová et al. (2016) evaluated the anti-diabetic effects of *Agrimonia eupatoria* L. and *C. altilis* infusions (200 mg/L) by tracking changes in serum glucose levels, α-glucosidase inhibitory activity, advanced glycation end products (AGEs) production, and butyrylcholinesterase activity. Although artichoke extract did not affect α -glucosidase activity, it lowered serum glucose levels and prevented the production of AGEs. Similarly, significant reductions in serum glucose and triglyceride concentrations were observed in rats with streptozotocin (STZ)-induced DM, after oral administration of ALE (200 and 400 mg/kg) (Heidarian and Soofiniya, 2011). Patients with metabolic syndrome were given ALE (1800 mg/daily) supplements for 12 weeks in a clinical experiment performed by Ebrahimi-Mameghani et al. (2018). Both insulin and the homeostasis model assessment of insulin resistance (HOMA-IR) levels dropped after ALE administration (Ebrahimi-Mameghani et al., 2018). These studies validated cardoon’s anti-diabetic action, which may be connected to its chlorogenic acid due to its glucose 6-phosphate-suppressing properties (Arion et al., 1997; Loi et al., 2013), and caffeoylquinic acid, which regulates α-glucosidase activity (Matsui et al., 2006). Two clinical studies are available so far on ALE treatment for hypercholesterolemia, insulin sensitivity, obesity, and excess weight (https://clinicaltrials.gov/ct2/results?cond=Cynara+cardunculus+&term=&cntry=&state=&city=&dist=&Search=Search).

Cardoon is a Mediterranean medicinal plant with bioactive substances such as dietary fibers, sesquiterpene lactones, and phenolic compounds. Further research is needed to understand the mechanism of action of cardoon metabolites fully. Although few clinical trials have been completed already, more studies are necessary to clarify the therapeutic dose and duration of treatment (Silva et al., 2022).

### 3.3 *Eclipta prostrata* (L.) L

*E. prostrata* (syn. *Eclipta alba* (L.) Hassak) is an essential medicinal plant in the tropical and subtropical regions (Rahman et al., 2011; Feng et al., 2019; Sanyal et al., 2022; Silalahi, 2022). A whole-plant methanol extract of *E. prostrata* was tested for its *in vitro* α-amylase inhibitory activities. With an IC_50_ value of 322.138 ± 0.025 μg/mL, the results showed moderate effectiveness in α-amylase inhibition, suggesting a possible anti-diabetic function (Alam and Sharma, 2020; Khan et al., 2020; Timalsina and Devkota, 2021). STZ (at a dose of 70 mg/kg of body weight) was injected into the peritoneum of rats in another experiment to induce DM. Wedelolactone from *E. prostrata* reduced the HbA1c level in diabetic rats (10.3% ± 0.72%) in the untreated group compared to the wedelolactone-treated diabetic group (7.2% ± 0.52%). Wedelolactone was able to reverse the abnormal increase in biochemical parameters, including urea and creatinine, seen in the STZ group. After 28 days of treatment with wedelolactone, the hepatic parameters, c-peptide and insulin secreted from β-cells, were shifted toward normal levels, confirming the effectiveness of the treatment (Shahab et al., 2018; Zhang et al., 2022). The α-glucosidase activity was inhibited via a dose-dependent mechanism by the *E. prostrata* ethanolic extract (10 μg/mL, enzyme activity was reduced by 88.6%, IC_50_ = 4.5 μg/mL) (Jaiswal et al., 2012). Moreover, when treated with this extract, pancreatic β-cells are also reported to be restored and regenerated (Hemalatha et al., 2006).

**FIGURE 4:**
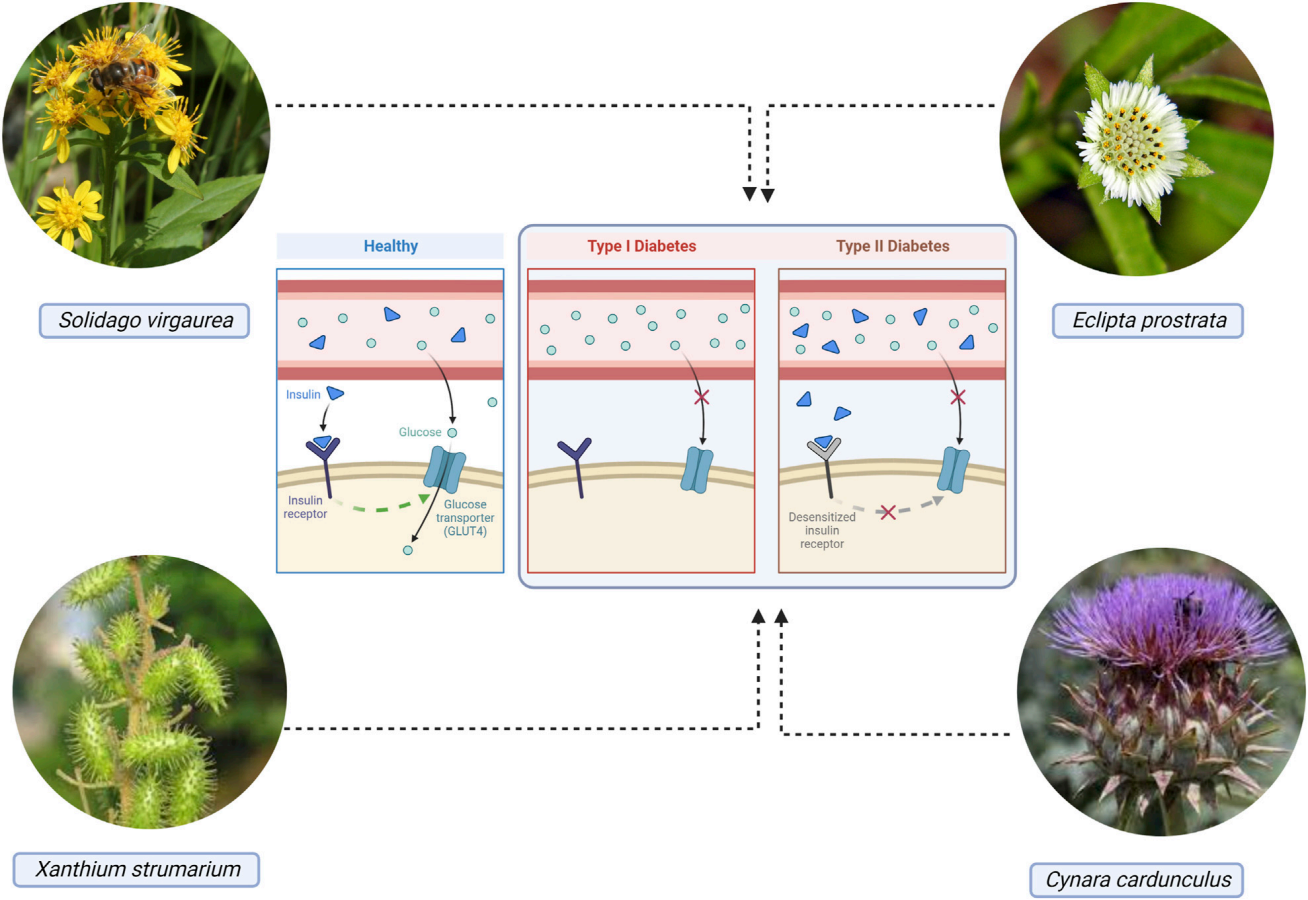
The antidiabetic effects of some plants from the Asteraceae family.

*E. prostrata* leaf suspension (2 and 4 g/kg) was orally tested for its antidiabetic effects (Figure 4) in rats with alloxan-induced DM. The extract normalized biochemical markers that were dramatically shifted due to DM. The anti-hyperglycemic action of *E. prostrata* in rats was demonstrated by an increase in liver hexokinase activity and a decrease in blood glucose level and glycosylated hemoglobin as a result of decreased activity of glucose-6 phosphatase and fructose-1,6-bisphosphatase (Ananthi et al., 2003; Yadav and Yadav, 2023). Although antidiabetic effects have been observed, the molecular mechanism behind these effects is poorly understood. Two clinical studies have been made available so far on the use of this extract for the treatment of chronic hepatitis B, HIV-I-infection, and AIDS (https://clinicaltrials.gov/ct2/results?cond=Eclipta+prostrata+&term=&cntry=&state=&city=&dist=&Search=Search). Still, future clinical studies about DM are necessary.

### 3.4 *Xanthium strumarium* L

*X. strumarium* is a common and well-known traditional Chinese herbal medicine, usually called Cang-Er-Zi, used for thousands of years in China. The dose-dependent, powerful hypoglycemic action of WEX (15 and 30 mg/kg, i.p.) was shown in normal rats by Kupiecki et al. (1974). Researchers studied the anti-diabetic effects of caffeic acid extracted from *X. strumarium* in 2000, using STZ-induced hyperglycemic and insulin-resistant rat models (Figure 3). Caffeic acid (0.5–3.0 mg/kg) reduced plasma glucose levels by enhancing glucose utilization (Hsu et al., 2000). In normal-glycemic and STZ-induced hyperglycemic rats, Narendiran et al. (2011) reported that MEXs at 100 and 200 mg/kg (p.o., for 30 days) had outstanding antidiabetic action. The competitive inhibition of aldose reductase (AR) and galactitol production in rat lenses by methyl-3, 5-di-O-caffeoylquinate was previously effective in preventing diabetic complications (Yoon et al., 2013). In addition, the CFMEXL also showed significant inhibitory activity against the α-glucosidase enzyme, with an IC_50_ of 72 μg/mL (Khuda et al., 2014). Similarly, additional research demonstrated that MEX possessed a potent α-glucosidase inhibitory activity with an IC_50_ value of 15.25 μg/mL (Ingawale et al., 2018). Clinical studies are warranted to validate the active constituents of this plant as a treatment for DM.

### 3.5 *Solidago virgaurea* L

*S. virgaurea* (European goldenrod) is among the most researched species in its genus and is widely used for the treatment of different diseases in Germany, the Czech Republic, Poland, the Russian Federation, Ukraine, Bulgaria, Romania, the Republic of Moldova, Korea, and China. In an alloxan-induced diabetic rat model, a hydroalcoholic extract of *S. virgaurea* caused a decline in glycemia, TNF-α, serum α-amylase activity, and pancreatic malondialdehyde, and a rise in serum insulin, hepatic glycogen, pancreatic superoxide dismutase (SOD), and catalase activities (Fursenco et al., 2020; Sanad et al., 2022; Zehra et al., 2023). Clinical studies are warranted to further validate the active constituents of this plant as a treatment for DM.

## 4 The role of asteraceae in diabetic wound healing

**FIGURE 5:**
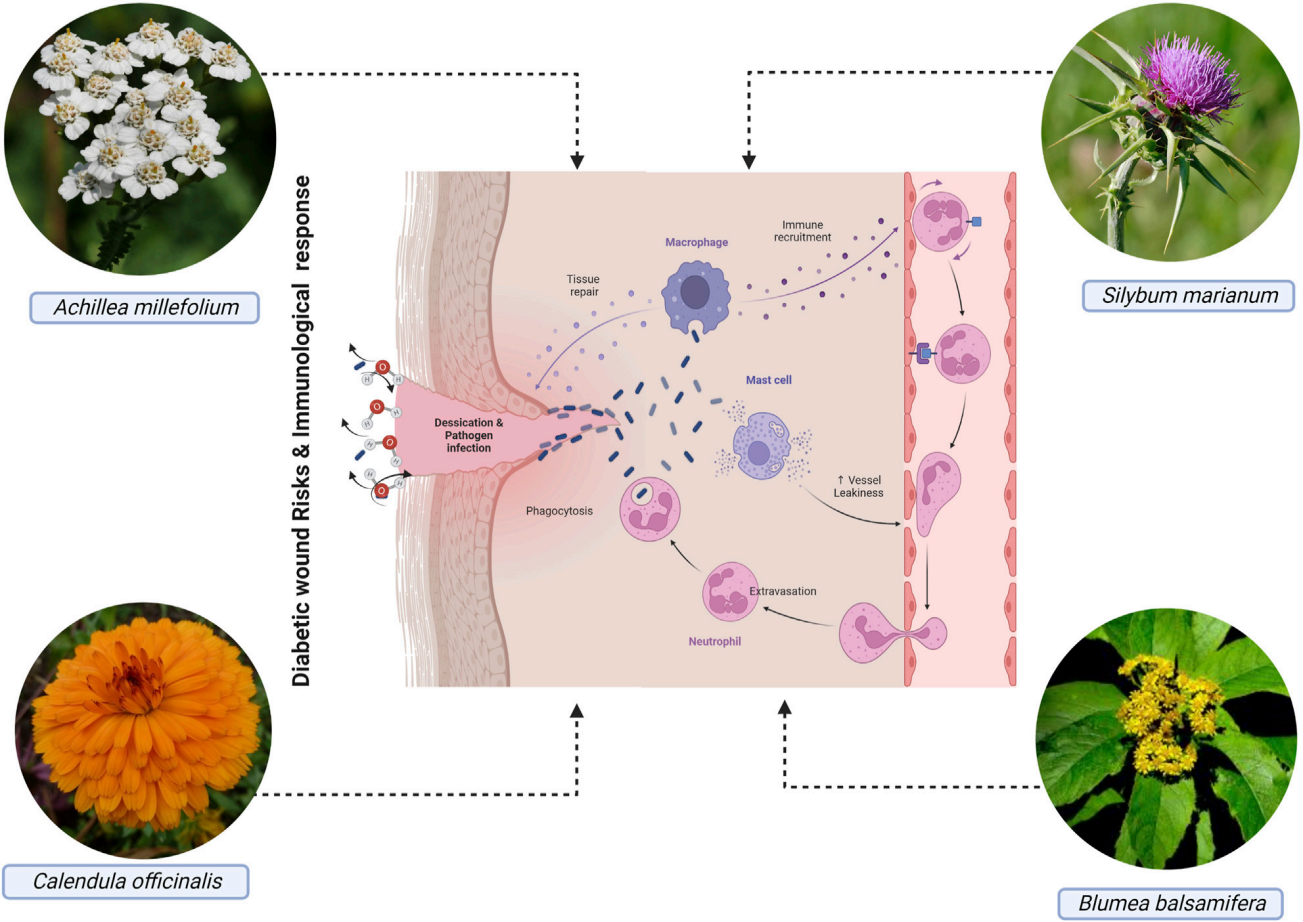
The wound healing activity of some plants from the Asteraceae family.

Large lesions and/or loss of function in the affected areas result from severe wounds. The presence of pathogens like *Staphylococcus aureus* and *Pseudomonas aeruginosa* makes wound care even more complicated, especially for people with chronic DM (Carvalho et al., 2018). Hence, novel wound-healing therapies must be developed without delay. Several members of the Asteraceae family are used as ethnomedicinal plants with wound-healing properties. The pharmacological qualities of these plants have been the basis of innovative and effective medicines, and the evidence collected from traditional medicine has paved the road for their development. Plants of the Asteraceae (Figure 5) have been extensively studied. Some of their pharmacological effects have been recognized, because of their widespread occurrence and ethnopharmacological significance, such as wound-healing, anti-inflammatory, antibacterial, antioxidant, and protozoa-fighting properties (Babotă et al., 2018; Özbilgin et al., 2018; George Kallivalappil and Kuttan, 2019; Ghaderi and Sonboli, 2019). These include *C. officinalis* (Dinda et al., 2016), and *Neurolaena lobata* (L.) R.Br. ex Cass, *Achillea asiatica S*erg (Dorjsembe et al., 2017), *Achyrocline alata* (Kunth) DC.(Pereira et al., 2017), *Acmella oleracea* (L.) R.K.Jansen (Maria-Ferreira et al., 2014), *Artemisia princeps* Pampanini (Lee et al., 2014), Blumea balsamifera (L.) DC. (Pang et al., 2017), *Pluchea indica* (L.) Less. (Sabri and Handayani, 2022), *Achyrocline satureioides* (Lam.) DC. (Alerico et al., 2015), *Artemisia asiatica* (Pamp.) Nakai ex Kitam (Park et al., 2008), *Artemisia argyi* H. Lév, and Vaniot. The proliferation of keratinocytes, and extracellular matrix remodeling, have been hypothesized to be essential for successful wound healing (Speroni et al., 2002; Rosa et al., 2014). Here, we discussed the most common antidiabetic plants of the Asteraceae, their bioactive compounds, and the mechanism of action presented in Table 2.

**TABLE 2:** Plants/compounds of the Asteraceae family that possess anti-diabetic and diabetic wound healing activities.

Plant name	Parts	Anti-diabetic activity		References
		Components	Activity related to DM & mechanism of action	
*Ageratum conyzoides* (L.) L	Lf	Kaempferol, precocene II	DM, CVD. Lowers blood glucose levels (BGL), increases plasma insulin levels (PIL), improves glucose uptake (GU)	Agbafor et al. (2015), Chandramohan et al. (2015), Kasali et al. (2021)
*Artemisia absinthium* L	Rh	α and β thujones, thujylalcohol, azulene, cadinene	Lowers BGL, activates adenosine mono-phosphate-activated protein kinase, and increases insulin sensitivity (IS)	Dabe and Kefale (2017), Bakun et al. (2021), Kasali et al. (2021)
*Artemisia capillaris* Thunb.	WP	-	DM, hepatitis, inflammation	Jang et al. (2015)
*Bindens pilosa* L	Rt	Cytopiloyne, apigenin, luteolin, kaempferol, quercetin	Lowers BGL and glycosylated hemoglobin. increases insulin expression and secretion from β- cells, stimulates glucose metabolism, increases IS to cells	Chang et al. (2013), Yang (2014), Xuan and Khanh (2016), Parveen and Ali (2019), Sangeetha (2019), El Barky (2020), Kasali et al. (2021)
*Cynara cardunculus* L	Wp	Alendronate (ALE), Chlorogenic acid, Caffeoylquinic acid Serum glucose	Antidiabetic activity. Significant reductions in triglyceride, and triglyceride concentrations, suppress glucose 6-phosphate, regulates α -glucosidase activity, α -glucosidase inhibitory activity, and butyrylcholinesterase activity	Arion et al. (1997), Matsui et al. (2006), Heidarian and Soofiniya (2011), Loi et al. (2013), Kuczmannová et al. (2016)
*Eclipta prostrata* (L.) L	Wp	Wedelolactone, liver hexokinase, glycosylated hemoglobin, carbon tetrachloride and terthiophenes	DM with other activities. Immunomodulatory activities, severe the abnormal increase in biochemical parameters including urea and creatinine, anti-hyperglycemic, decreased activity of glucose-6 phosphatase and fructose-1, 6-bisphosphatase, hepatoprotective activities, anti cancer activity	Ananthi et al. (2003), Jayathirtha and Mishra (2004), Thirumalai et al. (2011), Shahab et al. (2018), Alam and Sharma (2020), Wang et al. (2022)
*Lactuca sativa* L	Lf	-	Reduces BGL (diabetic), decreases creatinine, ALT, AST, and cholesterol levels	Chadchan et al. (2016), Ismail et al. (2019), Nabi and Abdullah (2020)
*Silybum marianum* (L.) Gaertn	Fd, Sd	-	Alcoholic induced liver diseases, DM2, wound healing	Anderson et al. (1994), Skottová and Krecman (1998), Hudaib et al. (2008), Jadhav and Upasani (2011), Aziz et al. (2016)
*Solidago virgaurea* L	Wp	Leiocarposide, polyphenolic acids, flavonoids, saponins	DM, anti-inflammatory, spasmolytic, and gastro-intestinal disorders	Móricz et al. (2016), Toiu et al. (2019), Móricz et al. (2020)
*Taraxacum officinale* (L.) Weber ex F.H.Wigg.	Wp	Taraxasterol (phytosterol), tetrahydroridentin B (sesquiterpene lactone), taraxacolide-β-D-glucoside (sesquiterpene lactone), Caffeic acid (phenolic acid)	DM, antihyperglycemic, anti-inflammatory, antimicrobial, hypolipidemic properties, anti-oxidative and immunostimulatory properties	Ceballos-Picot et al. (1996), Jones and Persaud (1998), Boden and Shulman (2002), Petlevski et al. (2003), Schütz et al. (2006), Arpadjan et al. (2008)
*Taraxacum platycarpum* Dahlst.	Wp	-	DM and hepatic diseases	Petlevski et al. (2003)
*Tithonia diversifolia* (Hemsl.) A.Gray	Fl	-	DM, anti-inflammatory	Baruah et al. (1979), Rüngeler et al. (1998), Tona et al. (1998), 1999 (2000)
*Vernonia amygdalina* Delile	Lf	Sobrerol, vernoamyoside E, luteolin, vitamin E	DM, gastrointestinal disorders, helminth infections. Lowers BGL and glycosylated hemoglobin levels, increases insulin secretion, enhances IS, and reduces oxidative stress	Asante et al. (2016), Balbi et al. (2018), Oyeyemi et al. (2018), Alara et al. (2019), Sangeetha (2019), Kasali et al. (2021)
*Xanthium strumarium* L	Wp	Caffeic acid, methyl-3, 5-di-O-caffeoylquinate, chlorogenic acid, sesquiterpene lactones	DM. Reduce PGL, Inhibition of aldose reductase (AR) and galactitol production inflammatory and analgesic activity	Seaman (1982), Chopra et al. (1986), Hsu et al. (2000), Chen et al. (2013), Yoon et al. (2013)

Plant name	Parts	Diabetic wound healing activity		References
		Model	Activity/mechanism	
*Achillea asiatica S*erg	Wp (ethanolic extract)	Sprague-Dawley rats (*in vivo*) and *in vitro* study with Hs68 fibroblasts	The extract enhanced healing by promotion of keratinocyte differentiation and motility and anti-inflammatory effects. It induced the expression of β-catenin, collagen, and keratinocyte differentiation markers	Dorjsembe et al. (2017)
*Achyrocline alata* (Kunth) DC.	Wp	Mice	The extract accelerated the healing by decreasing the initial inflammatory response and promoted re-epithelization and collagen remodeling	Pereira et al. (2017)
*Achyrocline satureioides* (Lam.) DC	Ap (ethanolic extract)	*In vitro*	Able to stimulate keratinocyte and fibroblast proliferation on the lower concentration	Alerico et al. (2015), Machado et al. (2020)
*Acmella oleracea* (L.) R.K.Jansen	Wp	Wistar Rats with gastric ulcers (*in vivo*)	Reduction of the treatment reduced gastric lesions due to its anti-inflammatory and antioxidant mechanism. It also induced cellular proliferation	Wu et al. (2008), Buragohain (2011), Abeysiri et al. (2013), Dubey et al. (2013), Maria-Ferreira et al. (2014), Gerbino et al. (2016)
*Ageratina pichinchensis* (Kunth) R.M. King and H. Rob	Ap (aqueous extract)	Murine models and clinical trials *in vivo*	Heals wounds in rats without inducing skin irritation	Romero-Cerecero et al. (2011)
*Artemisia argyi* H. Lév, and Vaniot	Wp, supplementation with olive and fish oil	Mice (*in vivo*)	Improves cutaneous wound healing in chronically stressed mice	Rosa et al. (2014)
*Artemisia asiatica* (Pamp.) Nakai ex Kitam	Wp (ethanol extract)	Mice	Efficient against gastric injuries induced by ethanol	Park et al. (2008)
*Artemisia campestris* L	Wp (aqueous extract)	Rat	Wound healing effect probably due to the anti-inflammatory, antibacterial and antioxidant activities of its phytochemical contents	Ghlissi et al. (2016)
*Artemisia princeps* Pampanini	Wp	Study with HUVEC1 (*in vitro*)	Jaceosidin promoted proliferation, migration, differentiation of human endothelia cells, and angiogenesis	Lee et al. (2014)
*Blumea balsamifera* (L.) DC.	Lf	Sprague-Dawley rats (*in vivo*)	The extract induced wound contraction, capillary regeneration, collagen deposition, and re-epithelization	Adebayo et al. (2010), Pang et al. (2017)
*Calendula officinalis* L	Wp (hydroalcoholic extract)	BALB/c mice and *in vitro* study with HDF2 (*in vivo*)	The extract is able to induce tissue granulation, proliferation, and cell migration	Lans et al. (2007), Dinda et al. (2015) (2016)
*Pluchea indica (L.) Less*	Ethanol extract	*In vivo* mice	Extract is capable to increase epithelial thickness	Sabri and Handayani (2022)
*Silybum marianum* (L.) Gaertn	Wp extract	Rat	Wound healing correlating with less redness, exudates and swelling	Hudaib et al. (2008), Aziz et al. (2016), Tabari et al. (2019)

Wp-Whole plant, Lf-Leaves, Fd- Flower head, St-Stem, Rt-Root, Ap- Aerial part, Fl-flower, Rh-Rhizome, Sd-Seed. DM; diabetes mellitus.

*B. balsamifera* is used in traditional Asian medicine as Ainaxiang, rich in volatile compounds such as L-borneol (a primary constituent), terpenoids, fatty acids, phenols, alcohols, aldehydes, ethers, ketones, pyridines, furans, and alkanes (de Boer and Cotingting, 2014). The volatile oil improved angiogenesis, collagen and epithelium deposition, and granular tissue development in injured Kun-Ming mice. This effect on wound healing and cell proliferation was linked to increased neuropeptide production (Pang et al., 2014a; Pang et al., 2014b). The volatile oil speeds up the recovery of Sprague-Dawley rats with burns by releasing growth factors and reducing pro-inflammatory cytokines (TNF and IL-1) (Fan et al., 2015). One week after the treatment with flavonoid-rich *B. balsamifera* leaf extract on Sprague-Dawley rat skin wounds, wound contraction, capillary regeneration, collagen deposition, and re-epithelialization occurred. These changes increased the expression of VEGF, TGF-β1, and CD68 in rat wound tissues.

*S. marianum*, also known as silymarin, is a wound-healing herb (Aziz et al., 2016). Increased epithelization and reduced inflammation are two benefits of silymarin, as observed in albino rats with excision wounds. Its extract has been shown to prevent oxidative damage in fibroblasts caused by lipopolysaccharides (Sharifi et al., 2012; Sharifi et al., 2013). In a similar vein, the flavonoid silibinin, which is derived from the silymarin herb, has been shown to speed up wound healing in rats by increasing the production of the extracellular matrix components stromelysin hydroxyproline, glycosaminoglycans, and collagen (Tabandeh et al., 2013). The cutaneous toxicity of nitrogen mustard is mitigated in mice by silibinin (Balszuweit et al., 2013); this further helps lower oxidative stress and inflammation (Jain et al., 2015). Another study found that a silibinin-based gel, applied topically for 14 days, helped wound healing by hastening epithelization, collagen production, and granulation tissue deposition (Samanta et al., 2016).

*C. officinalis* has been used in Europe as a wound treatment since the 13th century (Cioinac, 2016). Many cosmetic and personal care products include their components. In one study, an ethanolic extract from *C. officinalis* flowers and its dichloromethane and hexane fractions increased angiogenesis in embryonated eggs and rats with skin lesions. This effect on the vasculature was due to inflammatory cell infiltration and collagen deposition (Parente et al., 2012). *C. officinalis* tincture increased fibroblast proliferation and migration via the PI3K and FAK/Akt pathways. This extract included mostly flavonol glycosides (Dinda et al., 2015). Moreover, human keratinocytes treated with *C. officinalis* flower extracts (n-hexane and ethanolic extracts) boosted IL-8 and NF-kB expression and migratory cell capacity. The extract also inhibited collagenase action in human dermal fibroblasts. It is hypothesized that flavonoids and saponins are responsible for these effects (Nicolaus et al., 2017). The hydroglycolic extract of *C. officinalis* (Plenusdermax) enhanced wound epithelization, reducing healing time in venous leg ulcer patients (Ahmad et al., 2012; Buzzi et al., 2016). Another study found that low-intensity laser therapy combined with *C. officinalis* oil reduces lesions in diabetic foot ulcers (Carvalho et al., 2016).

Traditional medicine uses the *Achillea* genus for therapeutic purposes (Mohammadhosseini et al., 2017). *A. millefolium* is a herb native to Europe and Asia that has been utilized for over 3,000 years (Ali et al., 2017). Its anti-inflammatory, antioxidant, antifungal, and therapeutic activities are attributed to sesquiterpenes and phenolic compounds in this plant (Karamenderes and Apaydin, 2003; Fierascu et al., 2015). An *in vitro* study with human skin fibroblasts showed that the hydroalcoholic extract from the aerial parts of *A. millefolium* induces cell proliferation (Ghobadian et al., 2015). More recently, oil extracts from aerial parts of *A. millefolium* reduced skin irritation in healthy individuals (Tadić et al., 2017). *A. asiatica* (syn. of *A. millefolium* var. *Manshurica* Kitam), also called Mongolian yarrow, has therapeutic properties. *In vitro,* treatment of the aerial portions of this plant with Hs68 fibroblasts stimulated collagen synthesis via transformation of growth factor-mediated pathways. The same extract increased β-catenin, Akt, and keratinocyte differentiation markers, boosting keratinocyte differentiation and motility. Chlorogenic acid, apigenin-7-O-glucoside, and schaftoside are connected to the *A. asiatica* ethanolic extract’s therapeutic benefits (Dorjsembe et al., 2017).

*P. indica* is an evergreen shrub predominantly distributed in South Africa and Thailand. *P. indica* tea is widely recommended for promoting health in Southeast Asia. The findings suggest that ingesting this substance may potentially mitigate weight gain and hyperglycemia in rats fed a high-fat diet. It may be helpful in the treatment of hyperglycemia, dyslipidemia, obesity, and wound healing (Sirichaiwetchakoon et al., 2020; Sabri and Handayani, 2022). DM1 and DM2 are characterized by β-cell loss, and emerging evidence indicates that apoptosis is the mechanism by which β-cells are lost in both types. The ethanolic extracts of *P. indica* leaves were observed to decrease blood glucose levels *in vivo* (Nopparat et al., 2019). The leaf of *P. indica* has been observed to mitigate the severity of DM phenotypes induced by STZ. The untreated mice administered STZ exhibited elevated levels of hepatic malondialdehyde and reduced levels of superoxide dismutase and catalase. The administration of *P. indica* leaf extract at a dosage of 100 mg for 8 weeks resulted in the dissolution of the aforementioned symptoms (Nopparat et al., 2020). According to a search, *P. indica* tea can potentially mitigate hyperglycemia and dyslipidemia in individuals with pre-DM without causing any adverse effects on the kidneys, liver, or blood. The leaf of this plant can be utilized to produce a tea or herbal medication that may aid in preventing hyperglycemia and dyslipidemia (Sirichaiwetchakoon et al., 2021).

Another plant with ethnopharmacological relevance is *Artemisia princeps*. It is traditionally used to treat inflammatory-related diseases and has had its properties scientifically proven in various *in vitro* and *in vivo models* (Min et al., 2009). Jaceosidin is extracted from this plant, which has also been identified as the main constituent of another species, *Artemisia argyi,* with ethnomedicinal use as a healing agent (Li et al., 2018). It can inhibit the production of pro-inflammatory mediators such as TNF-α, IL-1β, and PGE2 (Min et al., 2009). *In vitro*, this flavonoid induces the proliferation, migration, and differentiation of human umbilical vascular endothelial cells. It also stimulates the formation of microvessels in rat aortic tissue, and this effect has been associated with the activation of VEGFR2/FAK/PI3K/AKT/NF-κB signaling pathways (Ali et al., 2017). Overall, all these studies suggest that jaceosidin is an interesting pro-angiogenic compound. Isosecotanapartholide, isolated from *A. princeps*, also has *in vitro* cell proliferating properties (Ali et al., 2017).

*Artemisia pichinchensis* is an ethnopharmacologically important plant in Mexico. Its pharmacological activities have been confirmed in murine models and clinical trials for diseases such as onychomycosis (Romero-Cerecero et al., 2009) and interdigital tinea pedis (Romero-Cerecero et al., 2012b, 2017). The first investigation demonstrated that *A. pichinchensis* aerial extract cures lesions in rats without causing skin irritation (Romero-Cerecero et al., 2011). Bio-guided purification showed that 7-O-(-D-glucopyranosyl)-galactin is the main component of *A. pichinchensis* that aids cell proliferation (Romero-Cerecero et al., 2013). Later, two extracts (aqueous and hexane) with standardized 7-O-(-D-glucopyranosyl)-galactin concentrations promoted skin lesion healing in diabetic rats (Romero-Cerecero et al., 2014). Human clinical investigations evaluated this plant’s therapeutic capabilities to treat chronic venous leg ulcers (Romero-Cerecero et al., 2012a). In another trial, diabetic patients with foot ulcers used a lotion containing *A. pichinchensis* extract that decreased healing time and lesion size, although no significant improvements were identified. The scientists attributed this to sample size, concluding that a larger clinical trial could prove *A. pichinchensis*’s effect on wound healing (Romero-Cerecero et al., 2015).

*Achyrocline* plants are crucial in Latin American traditional medicine (Bolson et al., 2015). In Rio Grande do Sul, Brazil, *A. satureioides* is utilized for healing. Extracts from the plant’s aerial portions trigger HaCaT keratinocyte growth (Alerico et al., 2015; Machado et al., 2020). Wistar rats showed efficient healing from treatment with *A. satureioides* essential oil applied to hydroxyethyl cellulose films (Yamane et al., 2016). *A. alata* and *A. satureioides* flower extracts were used to treat skin lesions in mice. Both extracts demonstrated positive benefits, but *A. alata* extract accelerated wound closure, resulting in faster healing (Balestrin et al., 2021, 2022; Salazar-Gómez and Alonso-Castro, 2022). Higher phenolic chemicals in *A. alata* caused this effect. Animals treated with *A. alata* extract had fewer mast cells at the site of inflammation, greater re-epithelization and granulation, and reduced initial inflammation (Pereira et al., 2017).

*A. oleracea* is a Brazilian plant used to heal skin and gastrointestinal ailments (da Rocha et al., 2018). *A. oleracea*’s rhamnogalacturonan inhibits ethanol-induced stomach ulcers in rats (Nascimento et al., 2013). Later studies showed that this chemical protects against acute (intraperitoneal) and chronic (oral) ethanol-induced damage. Rhamnogalacturonan improved gastric cell proliferation, mucus content, inflammation, and oxidative stress (Maria-Ferreira et al., 2014). Another study developed hydroxyethyl cellulose (HEC) films with *A. oleracea* ethanolic extract and *A. satureioides* essential oil. The HEC films containing these two plant components promoted wound healing in Wistar rats by increasing collagen deposition. α-Humulene and spilanthol were found to be responsible for the wound healing properties of these plants (Yamane et al., 2016).

Artemisia’s pharmacological potential has also been investigated in healing models. *A. asiatica* extract proved effective against ethanol-induced stomach injuries (Park et al., 2008). *A. argyi* repaired oral rat ulcers (Yin et al., 2017). Another study found that *Artemisia montana* (Nakai) Pamp essential oil promotes keratinocyte proliferation and collagen production. *In vivo,* tests demonstrated that *A. montana* essential oil enhances wound healing in rats (Yoon et al., 2014). *A. campestris* aqueous extract decreased inflammatory cell populations in the wound and promoted wound healing (Ghlissi et al., 2016).

## 5 Molecular antidiabetic phyto-compound database: DiaNat-DB

**FIGURE 6:**
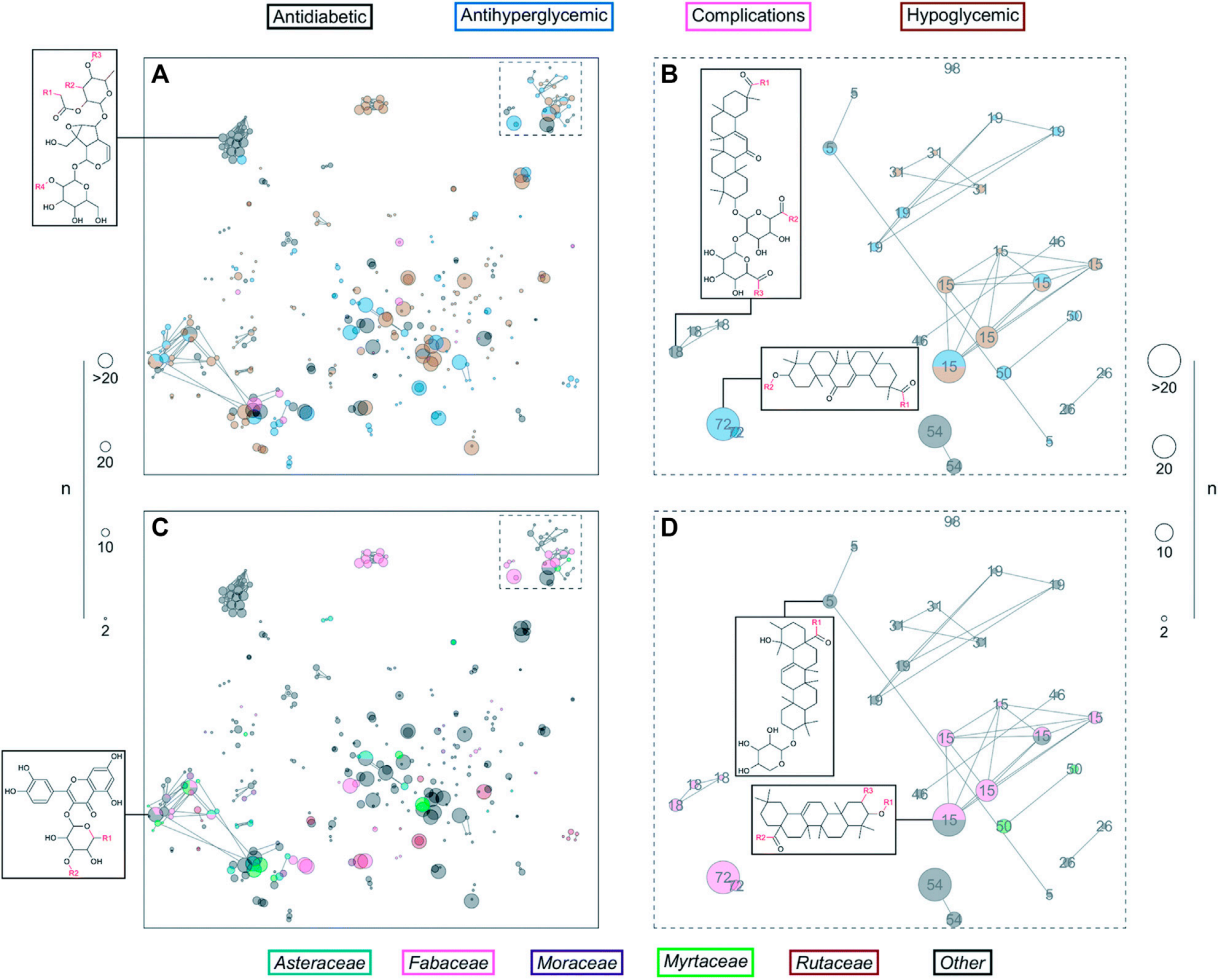
DiaNat-DB and ZINC15 analogs in a constellation diagram. The size of the dot shows the number of molecules mapped to the center. Colored dots represent DiaNat-DB compounds **(A**, **B)** based on the type of activity they are known to have, and **(C, D)** based on the plant family from which they were derived (Madariaga-Mazón et al., 2021). (CC-BY common attribute license).

DiaNat-DB is an original and one-of-a-kind database (first version) focusing on anti-diabetic natural products. The most recent version (the first one) includes 336 different compounds. An examination of the database’s features and structures revealed that the molecules contained within DiaNat-DB have, in general, characteristics like those of medicines. In addition, scaffolds are highlighted in the DiaNat-DB database because we could determine which structural scaffolds are present in antihyperglycemic and hypoglycemic actions by correlating the most common chemotypes with biological activity. This gave us insight into which compounds had these activities and were approachable for future drug development. In biochemical tests, the significant structural variety and complexity of the compounds contained in DiaNat-DB hint at the possibility (Figure 6) that the database could produce molecules with a high level of target selectivity. Finally, the hit expansion analysis enabled it to extend from 336 compounds to more than eight thousand analogs. These analogs constitute the starting points of lead optimization programs that are inspired by natural products. The general public can access DiaNat-DB which provides a significant direction in the integration of chemical and biological information for the discovery of natural anti-diabetic drugs. Compound databases are an essential component of chemo informatics as well as several other informatics-related disciplines that have significantly impacted the fields of chemistry, biology, and biomedical sciences (López-López et al., 2021; Madariaga-Mazón et al., 2021).

## 6 Role of asteraceae as dietary supplements/nutraceuticals to control DM

Since the link between nutrition, health, and happiness has been discovered, food is now seen as more than just a way to fill the stomach and ward against deficiencies; it is also considered a significant means by which people travel toward their health and happiness goals. With rising global prosperity, improved access to information on how to eat safely and healthily has increased overall life expectancy. In 2016, the WHO found that a high percentage of adults were overweight or obese. This frequency has increased among children and adolescents (WHO, 2018). Considering the current pandemic context, in which many people with chronic non-communicable diseases are at risk (Stefan et al., 2020), the dissemination and contribution to increasing the knowledge of important foods that improve health and satiety, which are easily accessible by the larger population who are living with low income, is crucial (Hobbs, 2020). Non-starchy tubers have been mostly overlooked for a long time because of low utilization and a lack of information about their cultivation worldwide, in contrast to the perennially popular starchy roots, tubers, and potatoes. Although this was the case previously, things have changed thanks to several studies and reports emphasizing the significance of prebiotic fibers in food, and tubers (Kocsis et al., 2007) and chicory (Pilon et al., 2006) have gained some commercial value.

In recent decades, there has been a rise in the use of nutraceuticals, defined as “dietary supplements or foods containing a broad kind of plant extracts, pure chemicals, and, more generally, functional compounds” (Colitti and Grasso, 2014). Nutraceuticals can address the immune system, obesity, aging, oxidative stress, *etc.* Non-starchy Asteraceae tubers contain prebiotic oligofructose and inulin (Flamm et al., 2001), while sunflower, safflower, chamomile, lettuce, and mug wort have broad commercial uses in the food and medicinal industries. All these can be utilized as dietary supplements and nutraceuticals for health benefits and the overall wellbeing of diabetic patients.

## 7 Recent advancements and the prospects of flavonoids in the management of DM

**FIGURE 7:**
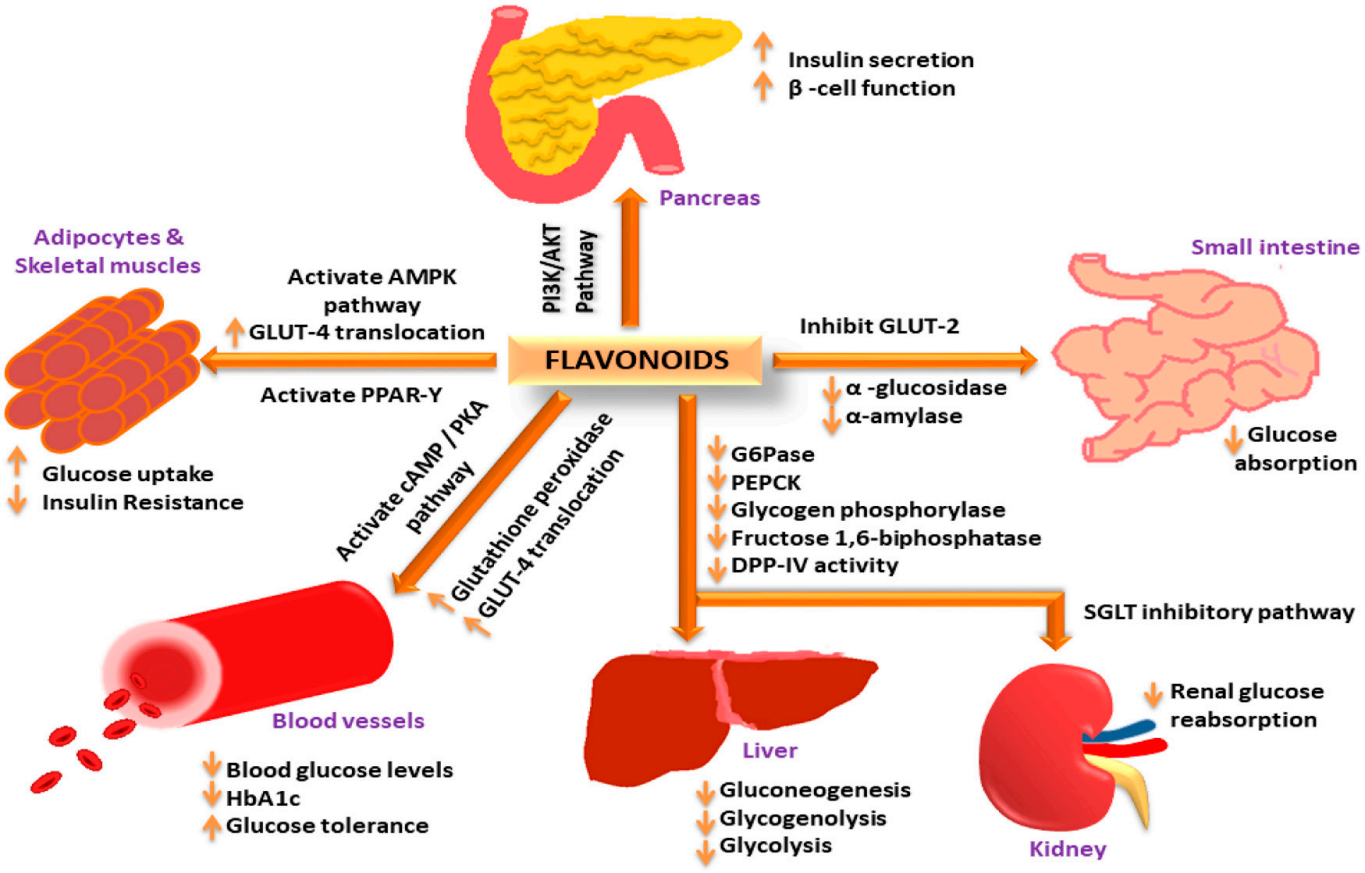
“Flavonoids exerting antidiabetic activity via different mechanistic pathways: Flavonoids increase insulin secretion and improve β-cell function via the PI3K/AKT signalling pathway; increase GLUT-4 translocation through AMPK activation to increase glucose uptake in adipose tissues and skeletal muscles; activate PPAR-γ expression to decrease insulin resistance; activate cAMP/PKA pathway to reduce blood glucose levels and improve glucose tolerance; increase glutathione peroxidase activity to reduce HbA1c levels; decrease glucose 6-phosphate, PEPCK, glycogen phosphorylase, fructose 1,6-biphosphatase and DPP-IV activity in the liver to decrease gluconeogenesis, glycogenolysis, and glycolysis; inhibit SGLT pathway in the kidney to decrease renal glucose reabsorption; inhibit GLUT-2, α-amylase, and α-glucosidase activity to reduce glucose absorption in the small intestine (Ansari et al., 2022a) (CC-BY common attribute license).

Flavonoids have been widely investigated as antidiabetic agents in cells, animals, human studies, and clinical trials. Two recent reviews published by Ansari and co-workers (Ansari et al., 2022a; 2022c) elaborately discussed flavonoids from products of natural origin as antidiabetic agents and the role of quercetin in the treatment and management of DM2. The authors examined how flavonoids exert their antidiabetic activity via different mechanistic pathways [see Figure 7] (Ansari et al., 2022a). As per the role of Asteraceae in DM control, what kind of flavonoids and their mechanistic pathway are debatable? Literature research revealed that dietary polyphenols prevent several diabetic-related complications, such as vascular dysfunction, nephropathy, retinopathy, neuropathy, cardiomyopathy, coronary diseases, and renal failure. There are reports available regarding polyphenols preventing and managing DM2 via insulin-dependent approaches.

As discussed previously, it is important to search for unique dietary polyphenols from the Asteraceae family to develop nutraceuticals that can be used to control DM. The common flavonoids most frequently isolated from the plants of this family are isoflavones, hydroxycinnamic acids, and caffeoylquinic acids.

A significant hypoglycemic effect was observed from dietary flavonoids via the inhibition of digestive enzymes (Xiao et al., 2013b; 2013a), regulation of intestinal microbiota (Gowd et al., 2019), and prevention of advanced glycation end-product formation (Xie and Chen, 2013), among other mechanisms. By controlling glucose metabolism, hepatic enzyme activities, and lipid profiles, flavonoids mitigate the development of DM and its complications (Al-Ishaq et al., 2019; Zhao et al., 2019). Flavonoids’ hydroxyl groups on rings B and C, as well as their unsaturated 2, 3-bond in conjugation with a 4-carbonyl group, help prevent the development of digestive enzymes and AGEs, but their methyl and glycosidic moieties are counterproductive (Xiao et al., 2013b, 2013a; Xie and Chen, 2013). However, it remains unclear how the structure of flavonoids in the diet contributes to their hypoglycemic effects in animals. Rarely has the antidiabetic action of flavonoid C-glycosides been reported in human or clinical studies.

Epidemiological studies have found strong evidence linking isoflavone consumption to a reduced risk of developing DM (Konishi et al., 2019). Isoflavones inhibit carbohydrate digestion and glucose uptake in the small intestine, leading to hypoglycemic effects in Goto-Kakizaki diabetic rats (Jin et al., 2018) and slowing the progression of renal interstitial fibrosis in diabetic nephropathic rats (Liu et al., 2018). By lowering blood sugar levels, protecting pancreatic cells from damage, reducing inflammation and oxidative stress, and blocking the Maillard process and arabinogalactan protein (AGPs) formation, isoflavones proved to be the most effective hypoglycemic agents (Chen et al., 2018a; 2018b). In addition, DM complications such as cardiovascular disease, nephropathy, retinopathy, neuropathy, and so on are all improved by puerarin. DM2 patients can benefit from taking genistein because it significantly reduces hyperglycemia (Fu et al., 2012; Rockwood et al., 2019), increases β-cell proliferation and decreases apoptosis (Gilbert and Liu, 2013), reduces cardiac inflammation and oxidative stress (Gupta et al., 2015), and strengthens bones (Odle et al., 2017; Wang et al., 2019).

*In vivo,* studies using STZ, alloxan, and high-fructose and high-fat diets to cause DM2 have shown that several major hydroxycinnamic acids, like ferulic acid, caffeic acid, and chlorogenic acid, have significant effects on blood sugar levels. The primary phenolic component, chlorogenic acid, is known to lessen the likelihood of developing DM2. Changes in the heart, liver, and metabolism were slowed by a diet high in chlorogenic acid (Bhandarkar et al., 2019). CGA was reported to improve both sensorineural auditory function and DM consequences in animal models (Hong et al., 2017; Mei et al., 2018). CGA can reduce fasting plasma glucose and hemoglobin A1c levels in mice with advanced DM (Jin et al., 2015). Still, little data suggests that chlorogenic acid-rich decaffeinated coffee can regulate animal blood sugar levels (Faraji, 2018). To a lesser extent, chlorogenic acid inhibited carbohydrate-digesting enzymes in mice (Yambe-Silavwe and Williamson, 2018) and had a modest effect on both fasting blood glucose and blood glucose levels in oral glucose tolerance tests (Igarashi and Takahashi, 2017). Diet-induced obese mice do not benefit from chlorogenic acid protection against metabolic syndrome characteristics when fed a high-fat diet (Mubarak et al., 2013).

The 5-caffeoylquinic acids have been shown to block both human salivary and pancreatic α-amylase, which suggests that they may help decrease glucose absorption in the intestines following a meal in overweight or diabetic individuals (Narita and Inouye, 2009; Nurul Islam et al., 2013). Although pharmacokinetic studies have shown that caffeoylquinic acids are poorly absorbed from the gastrointestinal system (Lafay et al., 2006), various investigations have been conducted to determine if caffeoylquinic acids can affect the metabolism of peripheral tissues. *In vitro* studies have shown that caffeoylquinic acids competitively block liver glucose-6-phosphatase, the enzyme responsible for the final reaction in gluconeogenesis. It was hypothesized (Arion et al., 1997; Hemmerle et al., 1997; Henry-Vitrac et al., 2010) that caffeoylquinic acids could exert hypoglycemic activity by suppressing hepatic glucose synthesis. One possible explanation for this finding is that the amount of caffeoylquinic acid in liver cells was too low to inhibit Glucose-6-phosphatase (Bassoli et al., 2015) effectively. Recent research has shown that chlorogenic acid, along with several caffeoylquinic acid derivatives, acts as a noncompetitive inhibitor of protein tyrosine phosphatase 1B, and of all the caffeoylquinic acids, it is the most powerful inhibitor, with a Ki value of around 15 micromolar (Chen et al., 2014; Zhang et al., 2018). These findings indicate that caffeoylquinic acids may help increase insulin sensitivity in people who are overweight or have DM2. Treatment with CGA increases glucose uptake in insulin-sensitive and insulin-resistant adipocytes, supporting the former theory (Alonso-Castro et al., 2008).

Long-term use of caffeoylquinic acids decreases blood sugar levels, boosts insulin responsiveness (Reis et al., 2018), lessens hepatic insulin resistance (Lecoultre et al., 2014), lowers serum lipids, and promotes weight loss (Martínez-López et al., 2019). Extracts high in caffeoylquinic acids are suggested by traditional medicine for the treatment of DM and obesity, and these findings corroborate the idea that caffeoylquinic acids profoundly affect human energy metabolism (Spínola and Castilho, 2017; Xie et al., 2019).

## 8 Dietary polyphenols and the gut microbiome for DM

Gut microbiota makeup is linked to chronic illnesses (e.g., obesity, DM2, CVD, and nonalcoholic steatohepatitis) and is a new factor in DM management. Larsen et al. (2010) compared the intestinal microbiota of DM2 and nondiabetic patients. *Firmicutes* and *Clostridia* were dramatically reduced, while β-proteobacteria, positively linked with plasma glucose, were highly concentrated in DM2 patients’ feces compared to those of non-diabetic people. Food affects gut microbial diversity. According to animal studies and clinical trials, polyphenols and polyphenol-rich diets lower DM2 and/or its consequences (Nie et al., 2019). Polyphenols and their microbial metabolites can improve glucose metabolism by modulating the gut microbiota (Cardona et al., 2013). Few studies have investigated how polyphenols in the diet might affect the microbiome of people with DM. It is important to remember that studies of gut microorganisms have been identified as a novel field of research for generating new techniques to combat DM2.

Recent proposals for *Akkermansia* as a new biomarker of intestinal health suggest that its regulation may have favorable impacts on DM2 (*Akkermansia* has been documented to have effects on obesity and gut inflammation) (Delzenne et al., 2015; Blandino et al., 2016). Polyphenols were also discovered to influence Akkermansia positively (Everard & Cani, 2013). One study found that polyphenols stimulate the development of *Bifidobacteria* (Bialonska et al., 2010). Another study found that samples from diabetic patients had fewer *Faecalibacterium prausnitzii* and butyrate-producing bacteria than those from healthy individuals (Qin et al., 2012). *Bacteroidetes*-to-*Firmicutes* ratios are positively associated with decreased glucose tolerance (Larsen et al., 2010). Flavonoid metabolites 3,4-dihydroxyphenylacetic acid and 3-hydroxyphenylpropionic acid may promote pancreatic β-cell survival and function (Fernández-Millán et al., 2014). Polyphenol microbial metabolites may influence bile acid synthesis, affecting host metabolism.

**FIGURE 8:**
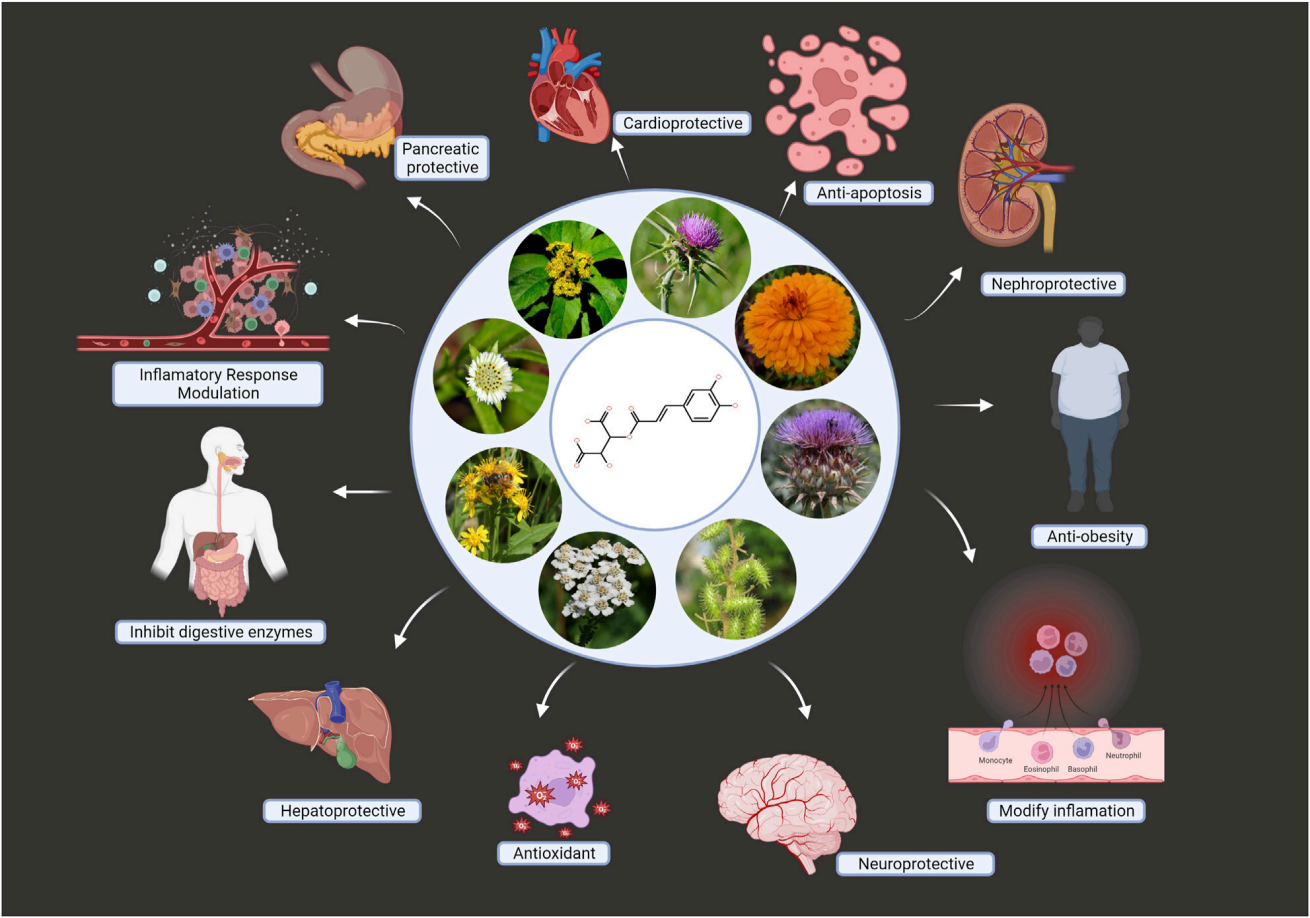
Asteraceae based dietary polyphenols in DM prevention. DM; diabetes mellitus.

In a nutshell, research has demonstrated a powerful interaction between polyphenols and the gut microbiota, both of which can protect against DM. Overall, the dietary polyphenols act on the gut microbiota and lower the ill-effects of DM in the following ways: (1) Polyphenols alter the gut microbiota by encouraging the expansion of good bacteria, including *Akkermansia*, *Bifidobacteria*, and *Faecalibacterium prausnitzii*; (2) bioactivity against DM is increased not only by polyphenols but also by their resulting microbial metabolites; and (3) more research is needed to determine how polyphenols and the microbes that metabolize them affect the microbiota in the digestive tract. Polyphenols reduce DM2 and diabetic consequences in animals (see Figure 8). It is unclear how DM2 affects polyphenol bioavailability and bioactivity, and understanding this mechanism will enhance clinical outcomes. The challenge is to produce enough excellent products to improve with the health of millions of people.

## 9 Challenges and opportunities of asteraceae medicinal plants in the management of DM

A wide variety of plants belonging to different families are available worldwide that are useful for treating many lifestyle-related disorders; this includes plants from the Asteraceae family too. There is no denying that plants from the Asteraceae family have demonstrated extensive use in traditional and modern medicinal systems due to their unique phytochemicals and bioactive compounds. This family has yielded many beneficial drugs (FDA approved) that have been and continue to be utilized for cancer, parasitic infections, and inflammation, among other diseases. However, despite their age-old use as traditional medicine, several compounds in this family lack scientific validation and, hence, have not been established or approved for treatment to date. The lack of sufficient documentation makes these plants unreliable for use. Also, the phytochemistry and essential oil content of many members of the Asteraceae are not well defined, and some plants have not even been studied yet.

### 9.1 Loss of traditional knowledge

The disappearance of traditional knowledge in developing countries is alarming. There should be preservation and awareness regarding the importance of indigenous knowledge and medicinal plants; a lot of valuable plants are becoming victims of deforestation and urbanization. As such, ethno-medicine and ethno-pharmacology should be introduced early in the curriculum of schools and colleges to make young people aware of ethnic practices. Government and non-government organizations should collaborate for the plantation and preservation of valuable medicinal plants, especially in developing countries. Since Asteraceae is a large family and many of the plants used by the indigenous peoples are not well explored, more work is needed for documentation to yield potent, commercially available botanically sourced antidiabetic drugs.

### 9.2 Safety

Safety concerns are another major issue with herbal drugs. The effects can be severe and detrimental if the appropriate dosage is not determined, as active compounds are toxic at high doses. The administration, distribution, metabolism, and excretion (ADME) of a compound that may act as a drug needs to be studied as it determines the bioavailability or fate of the compound; this stands true for all compounds (synthetic and herbal) that can be developed as drugs in the future. The FDA, United States, has designed a set of guidelines for all pharmaceutical companies and drug manufacturers to strictly adhere to for proper manufacturing, safety, and toxicity labeling. This ensures that all information is available to consumers to make an informed choice or purchase. However, several countries do not have appropriate regulations for quality control and risk assessment of bioactive compounds from natural sources. Coupled with the lack of consistency, misinformation between different countries across the globe can lead to disastrous effects and prevent the establishment of plant-based compounds as therapeutic products and nutraceuticals.

### 9.3 Bioassay guided purification

Extraction and characterization of bioactive compounds is a major challenge to drug discovery and development, even though high-end techniques, combinatorial chemistry, and high-throughput screening are well-developed. Most importantly, a minuscule number of “pure” compounds are obtained after lengthy, exhaustive procedures, which is insufficient for bioactivity screening. Though organic chemistry and synthesis can be applied to overcome this limitation, this process is not economically feasible. Also, there is a vast disparity in the activity of the natural compounds *in vivo* and *in vitro*; this can be attributed to the complex metabolic and synergistic pathways within the living systems that bring about their promising results *in vivo* but are extremely difficult and nearly impossible to replicate *in vitro* (Chakrabartty, 2022; Chakrabartty et al., 2022). As a result, drug discovery and the development of some potential and promising lead compounds take a backseat. Few studies are followed up by bioassay guided purification, so more research is necessary for scientific validation.

### 9.4 Lack of R & D on natural products in the pharmaceutical industries

Like many other families of medicinal plants, Asteraceae also show promising results for treating lifestyle-related disorders like DM and hypertension. However, the preliminary results do not highlight the actual underlying mechanisms of action of the different extracts and compounds. Hence, focused research is the need of the hour; extensive research is required on mechanisms, toxicity, risk assessment, safety, ADME, drug dosage, and formulation development. Such studies may attract the attention of big pharmaceutical companies to fund the subsequent research and commercialization of the potential drug leads from Asteraceae and all other medicinally important plants.

## 10 Conclusion and prospects

Lifestyle-related disorders have become commonplace in today’s world owing to the hectic and sedentary lifestyle, stress, and lack of awareness or consciousness of one’s health. Asteraceae is traditionally used worldwide to treat various diseases, including infections, wound healing, liver dysfunction, and other disorders. Complex secondary metabolites play a major role in bringing out these activities. Despite the excessive scientific studies in the search for new antidiabetic drugs, more work is needed to yield potent, commercially available drugs based on medicinal plants. Indeed, the scientific community and pharmaceutical companies have failed to provide the required attention to this plant family to explore therapeutic products, although a Nobel Prize has already been awarded. Although several plants from this family were extensively studied, some of the species important in traditional medicine have still hardly been studied for their bioactivity. Therefore, the present review aims to encourage in-depth exploration of different members of the Asteraceae family for the treatment of DM, guided by folk and traditional knowledge. The daisy family exerts antioxidant, hepatoprotective, vasodilation, antidiabetic, and wound healing effects, which further prevent major diseases like CVDs, liver cirrhosis, and DM. Most of these studies are still in the preliminary stages of *in vitro* and *in vivo* experiments, while clinical trials are lacking. No doubt, a few studies related to flavonoids and their mode of action in the treatment and management of DM are available, but pharmacokinetics/pharmacodynamics in laboratory animals and clinical trials are warranted to investigate their effects, including the mechanisms of action.

The information documented in this review article can serve as a pioneer for developing research initiatives directed at the exploration of the Asteraceae family at the forefront of developing a botanical drug to be used as a treatment for DM. With a shift towards plant-based and herbal products for nutrition and wellbeing, there is a high chance that consumers may easily opt for natural products for food and medicine in the long run.

## Figures and Tables

**Table:** 

**WEX**	Water extracts of fruit of *X. Strumarium*
**MEX**	Methanol extracts of fruits of *X. Strumarium*
**CFMEXL**	Chloroform fraction of methanol extracts of leaves of *X. Strumarium*
**SOD**	Superoxide dismutase
**CVD**	Cardio vascular disease
**WHO**	World health organization
**MD**	Mediterranean diet
**CT**	Computerized tomography
**MRI**	Magnetic resonance imaging
**VAT**	Vosceral adipose tissue
**BMI**	Body mass index
**TS**	Taraxasterol
**FOS**	Fructooligosaccharides
**CRA**	Chicory acid
**SEL**	Sesquiterpene lactones
**c-AMP**	Cyclic adenosine monophosphate
**SUR1**	Sulphonylurea receptor 1
**DM**	Diabetes mellitus
**DM1**	Type 1 Diabetes mellitus
**DM2**	Type 2 Diabetes mellitus
**IC**_**50**_	Half maximal inhibitory concentration
**ACE**	3-amino-9-ethylcarbazole
**AGE**	Advanced glycation end products
**ALE**	Artichoke leaf extract
**HOMA**	IR- homeostasis model assessment-estimated insulin resistance
**Hba1c**	Hemoglobin a1c
**TNF**	Tumor necrosis factor
**IL1**	Interleukin1
**VPGF**	Vascular endothelial growth factor
**TGFβ1**	Transforming growth factor beta 1
**CD68**	Cluster of Differentiation 68
**VEGFR2**	Vascular endothelial growth factor receptor 2
**FAK**	Focal adhesion kinase
**PI3K**	Plasma membrane-associated lipid kinases
**AKT**	Protein kinase B
**NF-κB**	Nuclear transcription factor kappa-B
**Syn**	Synonyms
